# Multifunctional nanozyme platforms in central nervous system therapies: from rational design to translational medicine

**DOI:** 10.7150/thno.136080

**Published:** 2026-07-29

**Authors:** Shufang Niu, Xiaoyin Liu, Ran Xu, An Zhu, Shihong Zhu, Fuheng Hu, Kunlun Ding, Siyi Li, Bingcan Zhu, Peize Liang, Siqi Zhang, Anqi Xiao, Kelong Fan, Zhiyong Zhang

**Affiliations:** 1 Department of Neurosurgery, West China Hospital, West China Medical School, Sichuan University, Chengdu 610041, Sichuan, China.; 2 National Engineering Research Center for Biomaterials, College of Biomedical Engineering, Sichuan University, Chengdu 610064, Sichuan, China.; 3 CAS Engineering Laboratory for Nanozyme, Key Laboratory of Biomacromolecules (CAS), CAS Center for Excellence in Biomacromolecules, Institute of Biophysics, Chinese Academy of Sciences, Beijing 100101, China.; 4 University of Chinese Academy of Sciences, Beijing 101408, China.; 5 Nanozyme Laboratory in Zhongyuan, Henan Academy of Innovations in Medical Science, Zhengzhou 451163, Henan, China.; 6 Department of Neurology, Beijing Geriatric Hospital, Beijing 100095, China.; 7 Neuromedicine Center, Beijing Geriatric Hospital, Beijing 100095, China.

**Keywords:** nanozymes, central nervous system disorders, neural regeneration, oxidative stress, neuroinflammation

## Abstract

Central nervous system (CNS) disorders—including ischemic stroke, traumatic brain/spinal cord injury, Parkinson's disease, and Alzheimer's disease—have long faced limitations in achieving functional recovery and disease-modifying therapies because of their complex pathophysiological mechanisms. Traditional therapies are often constrained by poor penetration across CNS barriers, limited participation in multiple pathological cascades, and insufficient persistence of therapeutic effects. Nanozymes are a class of nanomaterials with enzyme-like catalytic activity and tunable physicochemical properties. Not only do these nanozymes continuously scavenge reactive oxygen and nitrogen species in pathological environments through stable multi-enzyme synergistic effects by leveraging their abundant active sites, but also serve as multimodal therapeutic delivery platforms to achieve efficient drug delivery, opening up new avenues for neuroprotection and regenerative medicine. This review systematically examines the fundamental characteristics, classification systems, and functional design approaches of nanozymes, along with their potential for combined therapeutic strategies, including synergistic applications with drugs, hydrogels, genes, or cells. Additionally, it summarizes the latest advancements in neuroprotection and repair associated with CNS disorders. The review further analyzes current limitations and challenges related to clinical translation and offers insights into future research directions to enhance scientific knowledge and clinical applications in this significant field.

## 1. Introduction

Globally, neurological diseases such as Alzheimer's disease (AD), Parkinson's disease (PD), traumatic brain injury (TBI), intracerebral hemorrhage (ICH), spinal cord injury (SCI) and ischemic stroke (IS) have become the main contributors to mortality, disability, and disability-adjusted life years (DALYs). These conditions have brought a heavy burden to public health 1. A recent assessment of brain health and the burden of neurological diseases by the Global Burden of Disease Study shows that neurological diseases have cumulatively affected as many as 3.4 billion people in the world—accounting for 43% of the world's population—a figure that continues to climb with the aging of the global population. Among these diseases, the annual economic burden of specific diseases such as dementia alone is close to or more than a trillion dollars 2. In addition, in the past two or three decades, the failure rate of phase II/III clinical trials of central nervous system (CNS) drugs for neurodegenerative diseases such as AD has been high. Effective interventions that can truly prevent or significantly change the course of these diseases are still extremely limited 3. Fundamentally, these seemingly heterogeneous diseases share a self-amplifying pathological loop—oxidative damage and oxidative stress–inflammatory interactions driven by excessive reactive oxygen species (ROS); when the central nervous system encounters trauma, ischemia, hemorrhage, or when misfolded proteins accumulate, damaged neurons and axons quickly release high-mobility group box 1, adenosine triphosphate (ATP), heat shock proteins and other damage-associated molecular patterns (DAMPs). This activates microglia and astrocytes, whereby pro-inflammatory cytokines (*e.g.*, IL-1β, TNF-α, and IL-18) and ROS amplify the NLRP3 inflammasome signaling through positive feedback loops. This inflammatory cascade further induces the deposition of toxic proteins such as amyloid-β (Aβ), α-synuclein (α-syn) and myelin debris to form a closed loop of "inflammation–oxidation–protein". This leads to loss of synaptic plasticity, axonal transport disruption and neuronal death, eventually manifesting as irreversible functional defects, including memory loss, cognitive decline and motor disorders 4, 5.

However, at present, most drugs and biotherapies target a single pathway or mechanism, and it is difficult to simultaneously cross the blood–brain barrier (BBB) and the blood–spinal cord barrier (BSCB), continuously scavenge ROS, and remodel the pathological lesion microenvironment. Although endogenous antioxidant enzymes are the main defense against oxidative stress, their clinical translation for CNS applications as an exogenous therapeutic agent is subject to many restrictions, such as poor barrier penetration ability, short half-life in the body, susceptibility to proteolytic degradation and high preparation and storage costs 6, 7. This prompts the scientific community to seek functional alternatives. Since Gao *et al.* first reported in 2007 that iron oxide (Fe_3_O_4_) nanoparticles have inherent peroxidase (POD) activity and formally proposed the new concept of "nanozymes" 8, this emerging interdisciplinary field has rapidly developed. A broad range of nanomaterials—including precious metals, metal oxides, carbon-based materials and metal-organic frameworks (MOFs)—have been reported to mimic the activity of one or more antioxidant enzymes 9. Compared with natural enzymes, these materials have shown significant advantages in terms of stability, tunability, cost-effectiveness, and multifunctional integration, making them strong candidates for the next generation of "artificial enzymes" with great application potential.

With the advantages of long-lasting catalytic activity, excellent ROS-regulatory capacity and high stability in the physiological environment, nanozymes have been proven to have application value in many fields such as biosensing, antibacterial applications, acute kidney/lung injury treatment and tumor treatment 10. Among them, antioxidant nanozymes can dismutate superoxide anions (O_2_^•-^), decompose hydrogen peroxide (H_2_O_2_), and attenuate highly reactive species such as hydroxyl radicals (**•**OH) and peroxynitrite (ONOO^-^) through cascade redox reactions_,_ thus alleviating cell oxidative stress 11. In the past decade, this strategy has been rapidly extended to various rodent of SCI, IS, TBI, and PD. Relying on their multienzyme cascade catalysis mechanism, surface modification and targeted delivery ability, a variety of nanozymes pass through or bypass the BBB/BSCB to achieve lesion-specific accumulation, where they exert neuroprotective and repair-promoting effects by scavenging ROS, inhibiting neuroinflammation, suppressing glial scar formation, and promoting neurovascular remodeling and axonal regeneration, thus providing a new material-biological intervention model for the treatment of CNS disorders 12.

Despite the aforementioned clinical translational potential of nanozymes, existing review literature remains conceptually fragmented. While prior reviews have established classification systems for catalytic mechanisms and rational design strategies 9, 13, and have documented applications ranging from biosensing to tumor therapy 14, most still focus on single materials or specific diseases, failing to systematically integrate material engineering demands with the clinical translation requirements of distinct pathological stages. This review aims to bridge this gap. Its contribution lies not in expanding the scope of materials or diseases covered, but in establishing combination therapy as the central organizational principle and constructing a five-dimensional analytical framework encompassing material design, catalytic behavior, delivery strategy, disease-stage suitability, and translational feasibility.

**Figure 1:**
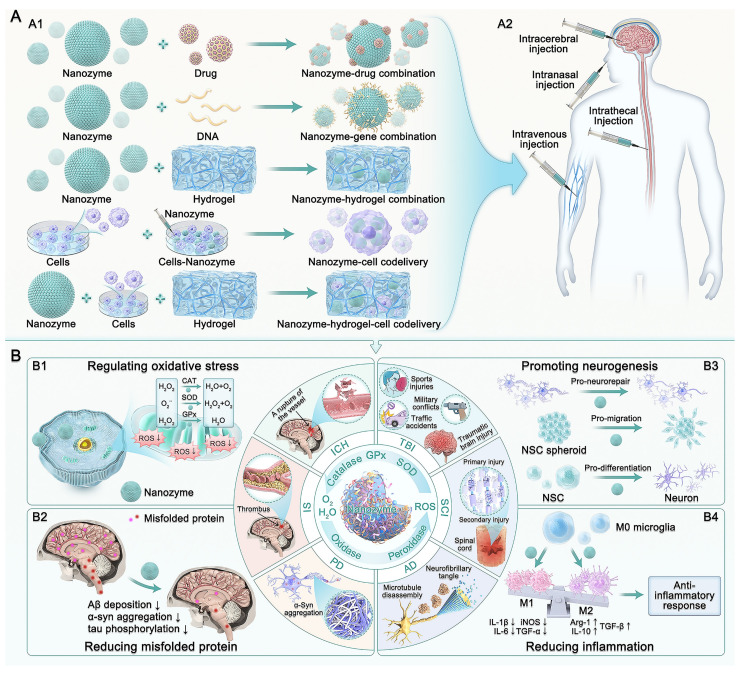
** Schematic illustration of nanozyme-based therapeutic strategies for CNS disorders. (A)** Nanozyme delivery systems and administration routes: **(A1)** integration of nanozymes with drugs, genes, hydrogels, or cells to construct versatile hybrid delivery platforms; **(A2)** multiple routes for CNS-targeted delivery, including intracerebral, intranasal, intrathecal, and intravenous administration. **(B)** Nanozymes exert multimodal therapeutic effects across diverse CNS pathologies (IS, ICH, TBI, SCI, AD, and PD) through four interrelated mechanisms: **(B1)** enzymatic ROS scavenging via SOD-, CAT-, and GPx-like activities to regulate oxidative stress; **(B2)** reduction of misfolded protein deposition; **(B3)** promotion of neurogenesis and functional neuronal differentiation; and **(B4)** reprogramming of microglia from the M1 phenotype to the M2 phenotype to attenuate neuroinflammation and foster a repair-conducive microenvironment. Abbreviations: Aβ: amyloid-β; AD: Alzheimer’s disease; Arg-1: arginase-1; BBB: blood–brain barrier; CAT: catalase; CNS: central nervous system; DNA: deoxyribonucleic acid; GPx: glutathione peroxidase; H_2_O_2_: hydrogen peroxide; ICH: intracerebral hemorrhage; IL: interleukin; iNOS: inducible nitric oxide synthase; IS: ischemic stroke; M0: resting microglia; M1: classically activated pro-inflammatory microglia; M2: alternatively activated anti-inflammatory microglia; NSC: neural stem cell; O_2_^•-^: superoxide anion; OXD: oxidase; PD: Parkinson’s disease; POD: peroxidase; ROS: reactive oxygen species; SCI: spinal cord injury; SOD: superoxide dismutase; tau: microtubule-associated protein tau; TBI: traumatic brain injury; TGF: transforming growth factor; α-syn: α-synuclein.

The framework integrates the research and development of nanozymes for the treatment of CNS disorders into five interdependent dimensions: (i) Material design: the composition and nanostructure determine the catalytic mechanism and the range of physically and chemically adjustable parameters that can be used for functional optimization. (ii) Catalytic behavior: the resulting enzymatic profile—whether reactive oxygen and nitrogen species (RONS) scavenging or stimulus-responsive catalytic switching—defines the therapeutic modulation capacity. (iii) Delivery strategy: BBB/BSCB penetration method and multi-functional platform engineering regulate the spatiotemporal biodistribution and on-site activation. (iv) Disease stage matching: The integration of the above engineering characteristics must be in line with specific pathophysiological situations, covering acute oxidative damage, subacute inflammatory remodeling and chronic protein lesion progression. (v) Translational feasibility: Each upstream design selection will have specific constraints on inter-batch consistency, long-term biological safety and clinical scalability, aspects that need to be confirmed by downstream verification. By examining these dimensions in turn, this review aims to clarify how the properties of basic materials are channeled through catalysis, delivery and disease-specific pathological contexts, so as to shape the treatment outcome and transformation trajectory (Figure 1).

## 2. Fundamental characteristics and classification of nanozymes

### 2.1 Fundamental characteristics

Nanozymes are a class of nanomaterials with enzyme-like catalytic activity, and typically possess nanoscale structural features (1–100 nm). They can catalyze specific chemical reactions under physiological conditions or extreme conditions, combining the high catalytic efficiency of natural enzymes and the stability of nanomaterials 14. With renewable active sites and multiple oxidation states on the surface, they can still maintain high-efficiency and stable catalytic ability even after multiple cycles 15. Compared with natural enzymes, nanozymes show better stability and activity, and are highly tolerant to temperature and pH fluctuations 16. Unlike traditional artificial enzyme, nanozymes integrate the inherent surface plasmon resonance, quantum size effect, high specific surface area, adjustable electron structure, and other characteristics of nanomaterials. This gives them a comprehensive advantage that traditional artificial enzymes cannot match in terms of atomic economy, catalytic multifunctionality and environmental tolerance 17. In recent years, researchers have achieved precise regulation of the electronic structure and microenvironment of the nanozyme activity center by constructing single-atom catalytic sites, regulating exposed crystal surfaces, designing heterogeneous interfaces, and using biological macromolecules (*e.g.*, proteins, nucleic acids, and peptides) for *in situ* coating. These methods enable them to selectively mimic a variety of activities in the oxidoreductase family, including oxidase (OXD), POD, catalase (CAT), glutathione peroxidase (GPx) and superoxide dismutase (SOD) 18. In addition, through surface functionalization, nanozymes can be endowed with a variety of functions such as targeted recognition, drug delivery and photothermal/magnetic response, so as to realize the integrated application of catalytic therapy, biological imaging and disease diagnosis. Their catalytic activity can be precisely regulated by parameters such as particle size, morphology, composition and surface charge, and can still maintain high stability under extreme pH, temperature or ionic strength conditions, showing excellent *in vitro*/*in vivo* adaptability and sustained intervention capability 19.

### 2.2 Classification and catalytic mechanisms of nanozymes

#### 2.2.1 Classification by catalytic function

The field of nanozymes has developed rapidly over the past decade, with more than 1,500 materials exhibiting enzyme-like catalytic activity reported worldwide 20. According to the classification framework of the Enzyme Commission (EC), natural enzymes are classified into seven classes, including oxidoreductases, transferases, hydrolases, lyases, isomerases, ligases, and translocases 13. Current research on nanozymes mainly focuses on oxidoreductase-like activities, while hydrolase-like and other activities are being increasingly explored.

The oxidoreductase-mimicking nanozymes are the most mature and widely used category in the field of nanozymes, accounting for more than 96% of all relevant studies. They cover a variety of subtypes such as OXD, POD, CAT, GPx, SOD. Typical representatives include Fe_3_O_4_, CeO_2_, Pt, CuO, MnO_2_ and related transition metal oxides or precious metal nanostructures. These materials efficiently mediate electron transfer through the reversible redox cycle of surface metal sites (*e.g.*, Fe^2+^/Fe^3+^, Ce^3+^/Ce^4+^, and Cu^+^/Cu^2+^). They have a simple and clear catalytic mechanism and readily characterized reaction kinetics, and can be seamlessly integrated with colorimetric sensing, tumor oxidative therapy, environmental pollutant degradation, and related applications 14, 21. More importantly, such nanozymes can mimic the catalytic processes of natural enzymes and bidirectionally regulate ROS; on the one hand, nanozymes with SOD or CAT-like activity can sequentially scavenge O_2_^•-^ and H_2_O_2_ through cascade reactions, blocking the formation of highly toxic radicals such as •OH, thereby significantly reducing oxidative stress during IS reperfusion injury and in the early stages of AD, and inhibiting tau hyperphosphorylation and Aβ aggregation; on the other hand, nanozymes possessing OXD and POD activity can catalyze the generation of highly oxidative species, such as •OH and O_2_^•^⁻, at tumor or bacterial infection sites, amplifying oxidative damage and promoting cell death 22, 23.

In contrast, nanozymes of other families—while exhibiting unique functional advantages in organophosphorus detoxification, site-specific protein cleavage, or stereospecific isomerization—remain largely confined to exploratory systems with significant limitations in catalytic efficiency, substrate selectivity, and mechanistic characterization 24. Given that CNS disorders are predominantly driven by RONS-mediated oxidative stress, inflammatory cascades, and mitochondrial dysfunction, this review focuses specifically on oxidoreductase-mimicking nanozymes (particularly SOD-, CAT-, and GPx-like systems) as the principal therapeutic modality for neurological applications.

#### 2.2.2 Classification by composition and structure

According to the composition and structure, nanozymes can be divided into four categories: carbon-based, metal-based, metal oxide-based and porous framework-based. Each system has shown significant differences in catalytic mechanism and functional regulation, and has been verified in many studies (Table 1).

Carbon-based nanozymes mainly include carbon nanotubes (CNTs), graphene, and carbon quantum dots (CQDs). Their sp^2^-conjugated carbon skeletons provide a conductive network and host diverse enzymatic active sites through surface defects, edge sites, and heteroatom doping, thereby enabling synergistic multi-enzyme catalysis 25, 26. For example, carbon quantum dots can catalyze the decomposition of H_2_O_2_ via surface hydroxyl and carboxyl groups, generating highly oxidative •OH and thereby exhibiting POD-like activity.

The catalytic activity of metal-based nanozymes with zero-valent metals (*e.g.*, Au, Ag, Pt and Pd) as the catalytic core originates from the local electromagnetic field enhancement induced by surface electronic structure, adsorption energy, exposure surface, coordination environment, local surface plasmon resonance in plasma metals, and particle size. Gold nanoparticles (AuNPs), gold nanoclusters (AuNCs), and mesoporous silica-coated gold nanoparticles (EMSN-AuNPs) are representative systems 27, 28. By tuning the particle size (usually <50 nm) and tailoring the surface chemical properties, metal-based nanozymes can integrate multiple enzymatic activities within a single nanostructure.

**Figure 2:**
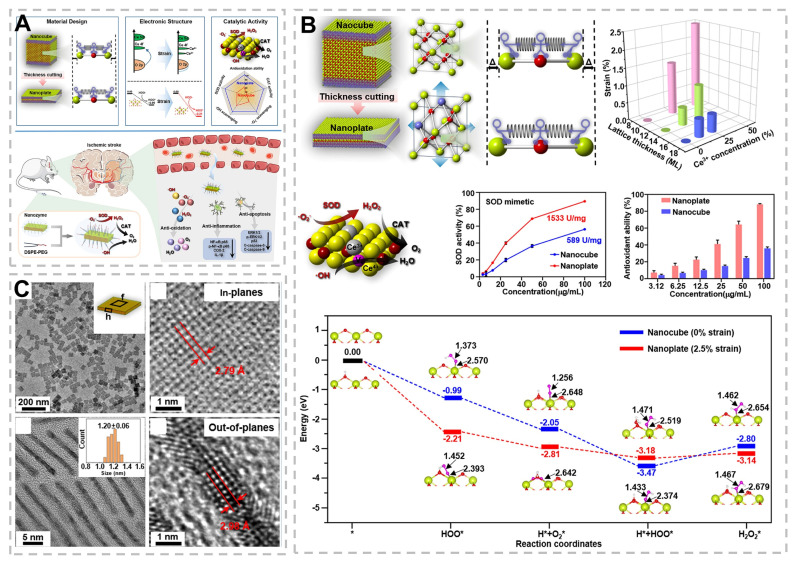
** Metal oxide-based nanozymes employ thickness modulation and lattice strain engineering to achieve coupled multienzyme activities in a single material. (A)** Schematic of strain-engineered ultrathin ceria nanoplates for IS therapy via coupled SOD/CAT-mimetic cascade antioxidation. **(B)** DFT predictions revealing the thickness–Ce^3+^–strain correlation, and experimental validation of the strain-enhanced multienzyme activities within a single nanostructure, and the corresponding DFT energy profiles elucidating the thermodynamic/kinetic origin of catalytic enhancement via reduced H_2_O_2_ formation barriers on strained surfaces. **(C)** HRTEM characterization confirming the tetragonal distortion in ~1.2 nm ultrathin nanoplates with ~3.0% in-plane and ~10.0% out-of-plane tensile strain, structurally underpinning the theoretical predictions and activity increase presented in (B). Adapted with permission from 30, copyright 2023, American Chemical Society. Abbreviations: CAT: catalase; DFT: density functional theory; HRTEM: high-resolution transmission electron microscopy; IS: ischemic stroke; SOD: superoxide dismutase.

Metal oxide-based nanozymes are mainly composed of metal oxides such as Fe_3_O_4_, CeO_2_, and CuO. The existence of redox active metal–oxygen vacancy pairs enables them to realize reversible switching between OXD/CAT/SOD-like activity in a wide pH range, providing a paradigm for intelligent responsive nanocatalytic platforms. CeO_2_ nanoparticles are a typical example: their mixed Ce^3+^/Ce^4+^ valence state gives them pH-dependent multienzyme activities, including OXD-, CAT-, and SOD-like activities 29. Notably, this intrinsic redox multifunctionality can be further amplified by thickness regulation: fabricating CeO_2_ into ultra-thin nanosheets of about 1.2 nm can induce intrinsic tensile strain, thus enhancing the covalency of Ce–O bonds and producing coordinatively unsaturated Ce sites with higher Ce^3+^ concentrations, thereby enhancing SOD-mimicking activity, CAT-mimicking activity and direct ROS clearance activity simultaneously within a single nanostructure (Figure 2) 30.

Porous framework-based nanozymes cover three types of crystalline porous materials: MOFs, covalent organic frameworks (COFs) and hydrogen-bonded organic frameworks (HOFs), which are assembled by metal–ligand coordination, strong covalent bonding and non-covalent hydrogen bonds respectively. Although the supramolecular connection chemistry of these compounds is different, they all have a highly ordered pore structure and adjustable chemical composition, so that they can achieve the precise construction of catalytic active sites for enzyme simulation. The microporous framework not only provides a microenvironment for substrate pre-concentration and diffusion regulation, but also enhances the interaction between the substrate and the active site to improve catalytic efficiency. In addition, the surface functional groups (including carboxylate and azide ligands) with the structure of MOFs can achieve high-density surface coupling, which is convenient for the connection of targeted groups for disease-specific treatment 31.

The systematic differences in the composition–structure–activity relationships of the four types of nanozyme families reveal their different catalytic essences and provide a basis for the rational design strategies described in the following sections. The systematic differences in these relationships also bring different but complementary translational characteristics to the treatment of the CNS. Carbon-based nanozymes perform well in biocompatibility and functional diversity, but the catalytic turnover rate is moderate, and there are concerns about long-term clearance. The metal-based systems have high intrinsic activity, but they face risks such as cost, large-scale production and chronic element accumulation. Metal oxide nanozymes have achieved a good balance between biocompatibility and versatile redox catalysis, but their activity remains sensitive to stoichiometric surface reconstruction and protein corona formation. Porous framework materials uniquely integrate ultra-high loading capacity and programmable biodegradability, but their physiological stability and manufacturing complexity still restrict clinical translation. In general, these trade-offs underscore that no single platform universally dominates; the optimal choice depends on the expected catalytic mechanism, the route of administration, and disease-specific pathological constraints.

## 3. Functional design strategies for nanozymes

**Figure 3:**
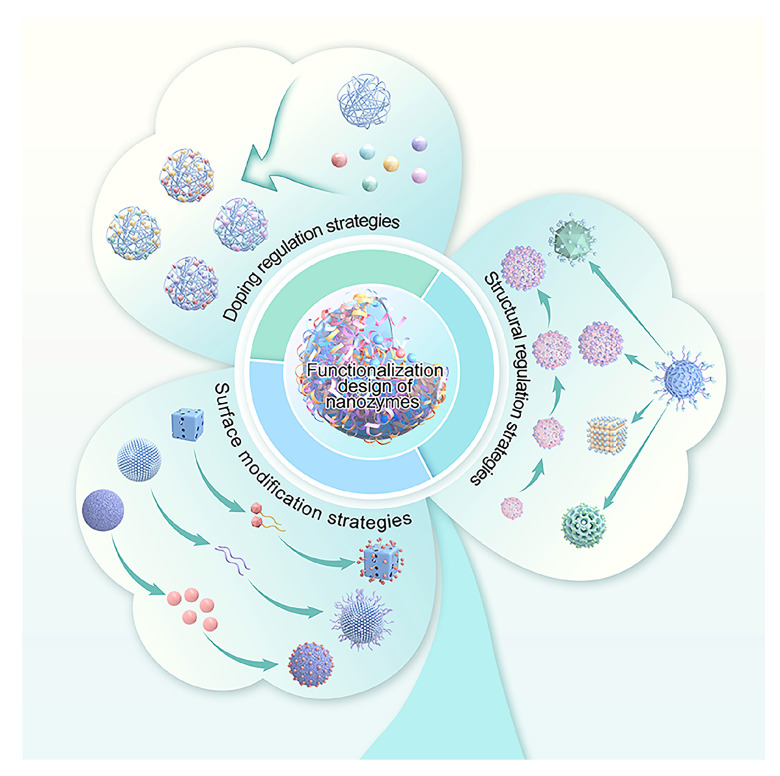
** Schematic illustration of regulation and functionalization strategies for nanozymes.** The composition and structure of nanozymes are optimized through doping regulation, surface modification, and functionalization design strategies to achieve precise control over their catalytic activity and biological functions.

Although nanozymes significantly outperform natural enzymes in terms of stability, controllability, and preparation costs, they still face bottlenecks in complex biological systems, including insufficient catalytic activity, low substrate specificity, and off-target risks arising from the spatiotemporal heterogeneity of ROS, pH, and enzyme profiles in disease microenvironments. To overcome these limitations, researchers have proposed biomimetic design strategies inspired by nature, in which the performance of a nanocatalyst can be optimized by mimicking key structural features of natural enzymes 32. On the one hand, introducing “cofactors” such as metal ions, ATP, and vitamins modulates surface electronic structures; on the other hand, active site engineering through amino acid modifications and single-atom M–N_4_, heteronuclear, or homonuclear bimetallic centers reshapes coordination geometries at the atomic scale (Figure 3). These strategies achieve synergistic optimization of multienzyme cascades, enantioselectivity, and microenvironmental responsiveness.

### 3.1 Structural regulation strategies

The structural design of therapeutic nanozymes requires a balance of four key features, including high catalytic activity, disease-specific microenvironment modulation, stability in pathological environments, and rapid renal clearance 33. Ultra-small materials (<5.5 nm) are the first choice because of their large specific surface area and their ability to pass through the glomerular basement membrane. However, simply reducing the particle size often sacrifices porosity and active site density. Porous framework-based nanozymes provide an alternative "size-structure" strategy: although their complete nanoframes usually exceed the kidney clearance threshold, their inherent biodegradability enables them to gradually decompose into clearable fragments. Meanwhile, the nanocrystalline fragments retain crystalline porosity, higher specific surface area and faster mass transfer rate 31, 34, 35. By anchoring single-atom or bimetallic centers (*e.g.*, M–N_4_, Fe–Fe, and Mn–Cu) in the topological network, the framework itself acts as a catalytic active site, rather than just acting as a carrier 36. The inherent biodegradability of these frames further enables them to gradually decompose and be excreted via the renal route after treatment, thus unifying high catalytic efficiency with rapid clearance 35.

For metal oxide-based nanozymes, thickness regulation and lattice strain engineering are powerful strategies for tuning their catalytic properties. For example, Liu *et al.* reported that ultra-thin ceria nanoplates (~1.2 nm) with intrinsic tensile strain (~3.0% in-plane and ~10.0% out-of-plane) exhibited a ~2.6-fold enhancement in SOD activity compared with strain-free nanocubes, reaching 1533 U/mg and approaching that of natural SOD; meanwhile, the CAT activity was increased by ~7.5-fold (Figure 2) 30. Beyond thickness regulation and lattice strain engineering, morphological control is also a critical dimension for modulating nanozyme performance. For example, compared with conventional morphologies, flower-like Mn_3_O_4_ nanozymes (Mnf) have a very high specific surface area and abundant exposed catalytic edges, displaying significantly enhanced SOD-, CAT-, and GPx-like activities, thereby highlighting the critical role of architecture-dependent catalysis in manganese oxide systems 37.

For metal-based nanozymes, size-dependent catalytic regulation is a major structural design strategy 27. At the nanoparticle scale (usually <50 nm), reducing the particle size can maximize the exposure of active surface atoms, thus increasing the density of the reachable catalytic sites. Therefore, catalytic properties are strongly dependent on particle size; for example, compared with free gold nanoparticles with a diameter of 13 nm, EMSN-coated gold nanoparticles with a diameter of 2.1 nm show significantly higher catalytic activity 28.

### 3.2 Doping strategies

Doping has been demonstrated to be an effective strategy for adjusting the structure and catalytic activity of nanozymes. Its essence lies in introducing foreign atoms (metals or nonmetals) to modulate the electronic structure, active site density, charge distribution, and reaction pathways of nanomaterials, thereby enhancing their enzyme-like catalytic performance.

#### 3.2.1 Metal atom doping

Metal atom doping can regulate the catalytic performance of nanozymes by changing the surface electron structure and energy band structure, which has been confirmed by many experiments and theoretical studies. The "metal-ligand" dimension of MOF provides a simple way for single-atom metal doping: the metal ions are isolated in the lattice node in advance, and then the atomic-level dispersed M–N_4_ active site can be generated through ligand-confined pyrolysis 38, 39. For example, Wu *et al.* constructed Mn–N_3_ and Mn–N_4_ single-atom nanozymes (SAzymes) by pyrolyzing Mn-doped ZIF-8 precursors under temperature-changing conditions, and confirmed that Mn–N_4_/SAzyme showed better POD- and glutathione oxidase (GSHOx) mimicking activities, highlighting the key role of precisely regulated coordination environment in determining enzymatic properties 40. Following this paradigm, Ye *et al.* reported an ultra-small platinum SAzyme, which integrates SOD/CAT-like activity and direct •OH clearance ability; in addition to redox regulation, Pt/SAE can also alleviate oxidative stress-driven pan-apoptosis and ferroptosis, while promoting macrophages to polarize from M1-like to M2-like phenotype, thus establishing SAzyme as a platform with dual functions of catalytic ROS clearance and innate immune reprogramming 41.

**Figure 4:**
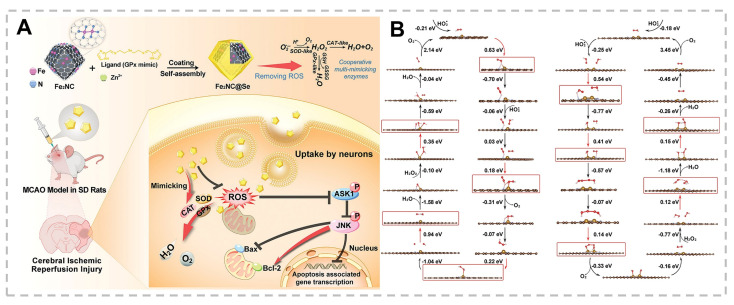
** Homonuclear bimetallic Fe_2_NC@Se nanoparticles for synergistic multienzyme cascade catalysis in cerebral ischemia–reperfusion injury therapy. (A)** “Precursor-preselected” synthesis of Fe_2_NC@Se and its therapeutic mechanism in MCAO rats. **(B)** DFT-calculated cascade pathways for Fe_1_NC (left) and Fe_2_NC (right), showing lower energy barriers (0.54 vs. 0.63 eV) and a four-order-of-magnitude rate enhancement via the synergistic effect of Fe–Fe. Adapted with permission from 42, copyright 2022, Wiley-VCH GmbH. Abbreviations: DFT: density functional theory; MCAO: middle cerebral artery occlusion.

Homonuclear bimetallic nanozymes feature two adjacent active sites of the same metal (*e.g.*, Fe–Fe, Mn–Mn, Cu–Cu) constructed on a single support. Leveraging metal–metal d-orbital coupling, they enable multielectron transfer and pathway regulation, achieving multienzyme cascade reactions or highly efficient O–O bond cleavage capabilities that are unattainable by single-metal sites. For instance, Tian *et al.* synthesized the Se-MOF-coated dual-Fe-atom nanozyme Fe_2_NC@Se via a “precursor-preselected” wet-chemical approach. The N_3_–Fe–Fe–N_3_ binuclear sites enable metal–metal d-orbital coupling-driven multielectron transfer, lowering the SOD/CAT cascade energy barriers and thus increasing the rate constant (Figure 4B). Together with the GPx-like activity of the Se-MOF shell, this establishes a genuine SOD–CAT–GPx triple-enzyme cascade. In a rat middle cerebral artery occlusion (MCAO) model, the neuronal uptake of Fe_2_NC@Se scavenges ROS through this cascade, suppressing the ASK1/JNK/Bax axis while upregulating antiapoptotic B-cell lymphoma 2 to protect against ischemia–reperfusion injury (Figure 4A) 42. In addition, as an essential trace element, copper is the core cofactor of natural antioxidant enzymes such as tyrosinase and copper-zinc superoxide dismutase (Cu/Zn-SOD). Liu *et al.* used a one-step method to construct a synergistic single-metal multivalency system with Cu_5.4_O nanoparticles (Cu_5.4_O USNPs), where Cu^0^ and Cu_2_O coexist in a two-phase state within the particles (mass ratio ≈ 3.4:1), forming a heterogeneous interface. The Cu^0^ domain decomposes H_2_O_2_ and O_2_^•-^, while the Cu_2_O domain clears •OH. Interface electron-hole separation broadens the ROS clearance spectrum and enhances stability, thereby enabling SOD-CAT-GPx triple-enzyme activity in the system. These ~4.5 nm nanoparticles can cross the glomerular basement membrane to achieve rapid renal clearance 43.

Heteronuclear dual-center nanozymes incorporate two distinct metals (*e.g.*, Mn-Cu, Fe-Co, and Ni-Cu) within a single nanoframework, each forming independent catalytic sites. Leveraging the difference in redox potential between heteronuclear metals and short distances (<1 nm), they achieve a substrate channeling effect, mimicking the catalytic logic of “differentiated metal collaboration” observed in natural cascade enzymes. For example, by using tetracarboxyphenylporphyrin (TCPP) as a ligand to simultaneously chelate Cu^2+^ and Mn^3+^/Mn^4+^, a 2.3 nm Cu-TCPP-Mn nanosheet was obtained via solvothermal synthesis and ultrasonicated to 20 nm in diameter. This structure forms an “MnN_4_-CuO_4_” adjacent bimetallic center at the atomic scale: the MnN_4_ site mimics Mn-SOD, rapidly disproportionating O_2_^•-^ to H_2_O_2_, while the CuO_4_ site mimics CAT, efficiently catalyzing H_2_O_2_ to H_2_O and O_2_. With an intercenter distance <1 nm, a substrate channel effect is achieved. The Michaelis constant (*K*_m_ = 34.7 mM) approaches that of native CAT (25 mM), and the catalytic constant (*k*_cat_) increases fourfold, indicating the occurrence of a true SOD-CAT cascade 44.

#### 3.2.2 Nonmetal atomic doping

Nonmetal doping is one of the core strategies to enhance the catalytic performance of nanozymes. Its essence is to use the electronegativity differences between atoms such as B, N, O, S, P and F and carbon or metal frameworks to induce localized charge rearrangement, energy band structure reconstruction and the generation of new active sites. This significantly enhances the efficiency of substrate adsorption and electron transfer. For example, undoped TiO_2_ nanoparticles showed minimal catalytic activity in the standard POD substrate systems. In contrast, TiN nanoparticles showed an order of magnitude increase in POD-like activity, and the catalytic efficiency was positively correlated with the nitrogen doping level 45.

In addition to nitrogen doping, phosphorus–oxygen dual-doped carbon nanosheets (POCNS) synthesized by a one-step pyrolysis method show significantly enhanced POD-like activity, wherein the synergistic dual doping of phosphorus and oxygen accelerates electron transfer and produces active sites with moderate free energy to activate H_2_O_2_ to generate •OH 46. The morphology of nanosheets further increases the number of exposed catalytic sites, thus realizing the sensitive colorimetric detection of H_2_O_2_ and glucose. Similarly, boron-doped graphdiyne (B-GDY), which is rich in ketonic carbonyl groups, can be used as a two-site carbon nanozyme. Its POD activity is about 4.2 times higher than that of the boron-free counterpart (GDY-700), because boron atoms and ketonic carbonyl (-C=O) together promote the adsorption and decomposition of H_2_O_2_, generating hydroxyl radicals 47. In addition, sulfur-nitrogen co-doped Fe–N–C nanozymes show excellent OXD-like activity and photothermal effect, indicating that heteroatom doping can give materials the characteristics of catalytic and photothermal function integration beyond single-enzyme mimicry 48.

In general, nonmetal doping has developed from empirical composition regulation to a rational electronic structure engineering paradigm. With the help of electronegativity differences, heteroatoms can induce directional charge distribution, reconstruct active site energy, and enable multifunctional catalytic behavior beyond single-enzyme mimicry.

### 3.3 Surface modification regulation strategies

Surface modification strategies, such as the use of macromolecules or small molecules for surface coating and functionalization, can significantly regulate the catalytic performance of nanozymes by changing the surface charge, electron density and substrate accessibility 49. Macromolecules (*e.g.*, bovine serum albumin (BSA), deoxyribonucleic acid (DNA), and polyethyleneimine (PEI)) can not only be used as templating and stabilizing agents to induce the formation of nanocatalyst morphology, but also exploit their pH sensitivity or sequence-programmable properties to control catalytic activation and inactivation. Small molecules (*e.g.*, citric acid and amino acids) enhance substrate adsorption through charge inversion or coordination interaction, effectively reducing the Michaelis constant (*K*_m_), thus improving the catalytic efficiency 50, 51. This tailorable, scalable, and biocompatible strategy enables the construction of interfacial nanocatalysts with integrated sensing and therapeutic functions.

For example, using acidic or basic amino acids to modify the surface of nanozymes can achieve precise regulation of the charge state of their surface, thus enhancing the affinity of the substrate. Aspartic acid-modified CuS nanozymes accumulate negative surface charges in a neutral environment, significantly improving the catalytic activity toward 3,3',5,5'-tetramethylbenzidine (TMB); in contrast, histidine-modified Fe_3_O_4_ nanozymes enhance H_2_O_2_ binding capacity through a positively charged microenvironment 51. In addition to simple amino acid coordination, metal–polyphenol coordination networks have emerged as a robust surface-engineering paradigm; for example, Zhu *et al.* constructed metal–polyphenol self-assembled nanodots (Fe@BDP NDs) via coordination-driven nanoprecipitation which integrate NIR-II fluorescence imaging with chemodynamic/photodynamic therapy-induced ferroptosis, demonstrating the versatility of phenolic surface chemistry in engineering multifunctional catalytic nanoplatforms 52. In contrast, protein adsorption can inhibit the catalytic activity of nanozymes, and the degree of inhibition varies according to the type of protein and nanozyme. Li *et al.* used physical adsorption to fix common proteins—including BSA, immunoglobulin G (IgG), avidin, and streptavidin—onto nanozyme surfaces of varying dimensions (0D–2D), covering Fe_3_O_4_ nanoparticles, Fe_3_O_4_@C nanoparticles, Fe_3_O_4_@C nanowires and graphene oxide nanosheets. The results show that the adsorption of these proteins inhibits the POD-like activity of nanocatalysts, and the inhibition level varies according to the type of protein and the type of nanocatalyst. Among them, BSA showed the most significant inhibitory effect on Fe_3_O_4_@C nanoparticles 49.

### 3.4 Machine learning and AI-aided design strategies

The catalytic activity of nanozymes is coordinated by multi-dimensional parameters such as metal type, valence state, size, morphology and surface chemistry, which makes it difficult to clarify the complex nonlinear structure–activity relationships through traditional trial and error methods.

To solve this problem, Wei *et al.* extracted 920 experimentally verified data points from more than 300 publications and built a standardized database covering 11 material and experimental descriptors. Uniform manifold approximation and projection dimensionality reduction analysis shows that the activities of the four enzyme types exhibit overlapping cluster characteristics; based on this, the team developed 14 fully connected deep neural network (DNN)-based models for enzyme type classification and quantitative activity regression, achieving *R*^2^ values of 0.66 and 0.80 for POD- and OXD-like activity prediction, respectively. Shapley additive explanations (SHAP) analysis reveals that metal type is the dominant factor regulating catalytic activity, indicating that ruthenium and manganese nanozymes exhibit optimal POD-like and OXD-like activities, respectively. These predictions have been experimentally verified by synthesizing polymetallic nanoparticles, and their *k*_cat_ trends are highly consistent with the model output 53. This work highlights the shift in the strategic paradigm of nanozyme research from "experiential induction and material specificity exploration" to "system modeling and cross-platform prediction".

At the same time, data-driven methods have begun to cope with the complexity of nanozyme design for biomedical applications. On the basis of preliminary work, Vinogradov *et al.* expanded the nanozyme database to 1,210 experimental samples, and incorporated the molecular descriptors of organic coatings and substrates into the machine learning framework for the first time. Through stacking ensemble model, they realized synchronous prediction of POD, OXD and CAT activity (*R*^2^ = 0.75 for *K*_m_; *R*^2^ = 0.77 for *V*_max_). SHAP analysis revealed that redox potential and electronegativity are the most influential electronic descriptors. The open access DiZyme platform and its large language model (LLM) auxiliary interface provide unprecedented tools for retrieving synthetic schemes and predicting multienzyme activities, which are expected to significantly accelerate the rational design of nanozymes that regulate RONS in the treatment of CNS disorders 54. Kan *et al.* used the gradient boosting regression (GBR) model to carry out *k*_cat_ prediction and obtained an* R^2^* of 0.95 on the test set. On this basis, they integrated the Nanozyme Copilot based on ChatGPT into the AI-ZYMES platform and established an integrated paradigm that unifies the prediction of nanozyme catalytic activity with the intelligent recommendation of the synthetic path 55.

Collectively, these studies have jointly established an integrated technology pipeline covering data mining, machine learning, interpretable analysis and intelligent design, and realized the quantitative exploration of the vast structure and composition space of nanozymes. This paradigm significantly accelerates the iterative cycle from material synthesis to functional verification, and promotes nanozyme design to transcend empirical intuition and enter the era of rational engineering.

## 4. Smart nanozymes

Smart nanozymes are defined as catalytic nanomaterials engineered by stimuli-responsive functional modules, which can realize the spatiotemporal control of enzyme activity—maintaining a "silent" state under physiological conditions, while becoming specifically activated at lesion sites with abnormal pH, elevated ROS or external energy field characteristics 56, 57. This dynamic capability is based on structural engineering, doping engineering and surface engineering discussed in the previous section, and transforms static physicochemical optimization into a dynamic catalytic gate control system. The continuous catalytic activity of nanozymes may lead to off-target toxicity in normal tissues. Therefore, intelligent design pursues the precise catalytic effect of time and space through two complementary paradigms: intrinsic activity switching and carrier-gated release 58. Activity switching involves the engineering design of the surface or lattice of the nanozyme, so that it can respond to endogenous signals (*e.g.*, pH and redox potential) or exogenous fields (*e.g.*, light and ultrasound) and switch the catalytic function reversibly. The carrier-gated release strategy encapsulates catalytic and stable nanozymes in a responsive carrier, which disintegrates, undergoes phase transition or opens pores under pathological triggers, thus regulating bioavailability without disturbing the active site structure. The combination of catalytic control at the molecular level and delivery control at the system level can achieve time accuracy and spatial specificity at the same time, and maximize the therapeutic effect of the lesion site while minimizing off-target toxicity.

### 4.1 Activity-switchable nanozymes

In biomedical applications, the continuous catalytic activity of nanozymes may pose a potential threat to normal tissues. In response to this problem, researchers have designed stimuli-responsive nanozymes capable of modulating their activity, thereby enabling precise spatiotemporal regulation of catalytic processes.

pH-responsive nanozymes use the acidic microenvironment of disease sites (such as tumors and inflammatory tissues) to realize the opening or closing of their activity. Based on this principle, Liu* et al.* constructed TiN nanozymes, in which nitrogen doping gives them strong POD-like activity, which can efficiently catalyze the conversion of H_2_O_2_ into •OH in an acidic tumor microenvironment (pH ≈ 6.0). Under 808 nm near-infrared irradiation, surface plasmon resonance is triggered, and the photothermal effect and hot-electron release are produced at the same time. The two simultaneously reduce the reaction energy barrier and instantly amplify the POD activity. In the absence of acid and light, their activity is negligible, thus forming an "acid-light" dual-control switch to minimize the potential damage to normal tissues 45. The cerium-enriched copper nanozyme developed by Lin *et al.* removes its PEG shell, reverses the surface charge in the weakly acidic tumor microenvironment, and precisely "unlocks" cell uptake. Subsequently, it releases Cu^2+^ and the prodrug disulfiram (DSF) through lysosome degradation, thereby achieving tumor-specific "nontoxic-to-toxic" transformation and cascade treatment 59.

Light-responsive nanozymes realize the dynamic regulation of enzyme activity through light irradiation. Representative categories include metal-based nanozymes, carbon-based nanozymes and MOFs. For example, oxygen/nitrogen co-doped carbon quantum dots (O/N-CQDs) can induce the polarization of surface quinoid nitrogen under visible light irradiation, thus triggering the reversible conversion of its activity from POD-like (producing ROS) to CAT-like (removing ROS). When the light is turned off, the polarization effect subsides and the initial activity is restored, thus realizing the intelligent switching between reactive oxygen generation and removal 60. Similarly, Zhang *et al.* combined graphite-phase carbon nitride (g-C_3_N_4_) with KOH and KCl to construct a metal-free dual-functional nanozyme (AKCN). The material can simulate the activity of glucose oxidase (GOx) under visible light and catalyze glucose oxidation to produce H_2_O_2_; in a dark environment, it then uses horseradish peroxidase (HRP)-like activity to oxidize the chromogenic substrate TMB to produce blue products, thus realizing rapid colorimetric detection of glucose 61.

Leveraging the merits of deep penetration, precise focusing, and noninvasive tissue stimulation, sonodynamic therapy (SDT) has become a frontier strategy for activating nanocatalytic systems in deep lesions. For example, Bai *et al.* fabricated ZnO@GDY nanorods by conformally coating graphdiyne (GDY) nanosheets on ZnO nanorods; under 1 MHz ultrasound excitation, the piezoelectric ZnO core produced a built-in electric field that accelerated carrier separation, resulting in a 7.2-fold increase in POD-like activity. The process demands neither light nor elevated temperature—activation is achieved purely by mechanical force (ultrasound), endowing it with noninvasiveness and outstanding controllability 62. Furthermore, efficient coupling of organic sonosensitizers with inorganic nanocarriers enables the construction of an *in situ* “silent-activated” enzyme-catalytic platform that achieves disease site-specific therapy. The “ultrasound-switchable nanozyme” strategy proposed by Sun *et al.* employs Pd@Pt nanoplates as the matrix, covalently modified with PEG-bridged meso-tetra(4-carboxyphenyl) Porphine (T790), to construct the Pd@Pt-T790 platform. T790 coating blocks Pd@Pt access to H_2_O_2_, keeping enzyme activity “silent” under physiological conditions and minimizing toxicity to normal tissues. After nanoparticles accumulate at infection sites and are exposed to ultrasound (US) irradiation, sonication-induced cavitation restores their catalytic function, resulting in the decomposition of endogenous H_2_O_2_ *in situ* to generate O_2_. This relieves hypoxia and increases SDT-ROS production, effectively overcoming the limited efficacy of conventional SDT in hypoxic environments 63.

In summary, the development of activity-switchable nanozymes provides a highly controllable platform for precise treatment and real-time diagnosis. These intelligent systems are driven by environmental clues such as pH or external fields such as light and ultrasound, and strictly perform their catalytic functions on demand. This spatiotemporal accuracy fundamentally minimizes off-target toxicity and provides a way for targeted clinical treatment and intelligent, low-cost biological sensing devices with great clinical prospects.

### 4.2 Nanozymes in stimuli-responsive release carriers

Smart nanozyme design can also be realized through a stimuli-responsive carrier system, which contains nanozymes with catalytic activity to achieve on-demand release. Under this paradigm, nanozymes maintain stable intrinsic activity, but their bioavailability is regulated by carrier disassembly, pore opening, or conformational transformation. These responses are triggered by environmental or external signals (such as pH, temperature, light, redox potential or enzyme). This carrier-mediated strategy decouples the catalytic function from the spatiotemporal deployment and operates on the delivery system scale to control the therapeutic dose.

To investigate the impact of photothermal effects on nanocatalytic therapy, Zou *et al.* embedded iridium oxide nanozymes (IrO_x_) in thermosensitive phase change materials (PCMs). Near-infrared light irradiation melted the PCMs, triggering the release of nanozymes. This responsive design enables precise nanozyme delivery and activation under specific conditions, minimizing damage to healthy tissues 64. In addition, this nanoencapsulation technology can effectively increase nanoparticle stability and prolong the systemic circulation time by minimizing interactions with the immune system, thereby improving therapeutic efficacy and reducing potential damage to healthy tissues 65.

While thermosensitive phase-change materials exemplify elegant single-input control, the pathological microenvironment of neural trauma presents multiple concurrent cues—including acidosis, redox imbalance, and enzymatic dysregulation—that invite more sophisticated carrier architectures capable of Boolean or sequential logic operations. In addition to this paradigm, the incorporation of ZnO-decorated ZIF-8 nanozymes into an injectable PVA-Alg hydrogel (ZnO-ZIF8@H) provides a dual-gated delivery platform that integrates endogenous acidosis sensing with external photothermal actuation 66. In the ischemic-hypoxic lesion of SCI, the ZIF-8 MOF scaffold underwent a pH-responsive structural collapse, whereas near-infrared irradiation (808 nm, 2.5 W/cm^2^) independently drove localized hyperthermia to 42.4 °C with a photothermal conversion efficiency of 46.6%; the synergistic operation of these orthogonal triggers confined Zn^2+^ release to the exact spatiotemporal coordinates where ROS accumulation and mitochondrial dysfunction coexisted, thereby enabling ferroptosis suppression, neuronal apoptosis rescue, and functional motor recovery (BMS score: 2 to 7 at 28 days) without systemic toxicity.

In summary, the strategies of switchable activity and carrier-gated release operate at fundamentally different length scales: the former enables instantaneous toggling of catalytic functions within the nanozyme active site, whereas the latter achieves targeted burst release or sustained exposure of preformed nanozymes through carrier disintegration. By integrating these two paradigms—activity gating at the molecular level and release gating at the system level—the intelligent nanozyme platform achieves time accuracy and spatial specificity at the same time.

Sections 3 and 4 systematically explain how structural regulation, doping engineering and stimulus-responsive design can optimize the catalytic behavior of nanozymes based on their intrinsic material properties. However, in CNS, the physical barrier of the BBB/BSCB, the multi-stage pathological progression, and the intertwining of oxidative stress–inflammation–regeneration cascade reaction make it difficult for single catalytic intervention to achieve barrier penetration, microenvironmental reshaping and functional repair at the same time in a narrow treatment window. Therefore, the engineered nanozyme platform must be combined with drugs, hydrogels, nucleic acid or cell therapy to close the loop between catalytic behavior and delivery strategy: nanozymes restore the redox balance at the lesion site through ROS scavenging, thus creating a treatment window for follow-up treatment; hydrogels realize local retention and continuous release; nucleic acid modules silence pathogenic signals; stem cell co-delivery reconstructs the neuromicroenvironment. The following chapters will systematically discuss how these combination strategies can establish a spatiotemporally controllable multimodal synergistic intervention system based on the aforementioned material–catalysis–delivery framework.

In addition to the application of the central nervous system, microenvironment-adaptive nanozymes have become an integrated platform for full-spectrum infectious chronic wound repair. Take the metal–polyphenol-coordinated system (*e.g.*, F@Gala) as an example. These nanozymes use pH-switchable catalytic cascade—exert POD-like activity under acidic conditions to kill bacteria, and exert catalase/superoxide dismutase-like functions in a neutral environment to alleviate oxidative stress. At the same time, the released biologically active ligands (*e.g.*, galangin) drive macrophage polarization from M1 phenotype to M2 phenotype, and simultaneously coordinate antibacterial defense, inflammation resolution and tissue regeneration 67. The integration of stimulus-responsive nanocatalysis and immune microenvironment regulation highlights the extensive translational value in various chronic inflammatory pathologies.

## 5. Nanozyme-based combination therapeutic strategies

Neurological diseases are often accompanied by oxidative stress imbalance, limited drug permeability and impaired regenerative ability, which makes it difficult for a single therapy to solve all problems at the same time. Therefore, the combined treatment strategy based on nanozymes integrates multimodal intervention methods (Table 2): first, nanozymes remove the RONS of the focal site to restore the redox balance, and at the same time deliver the drug to achieve synergistic catalytic drug release; then, the drug is embedded in the injectable hydrogel, forming a sustained-release reservoir to extend the treatment window. In addition, the platform can be supplemented with gene modules to silence drug resistance or pro-inflammatory signals, or codelivered with stem cells/immune cells to promote tissue repair. This method integrates catalytic therapy, drug delivery, nucleic acid therapy and cell therapy into a unified, spatiotemporally controllable therapeutic cascade.

### 5.1 Nanozyme–drug combinations

The pathogenesis of CNS disorders is highly complex, and monotherapy is often limited in efficacy and hindered by the BBB. Nanozymes offer multienzyme activity, ROS scavenging, inflammation modulation, and drug-loading capacity via functionalization. Combining nanozymes with drugs enables multitarget synergistic intervention, improves drug delivery, enhances neuroprotection and repair, and reduces side effects, providing an innovative therapeutic strategy for CNS disorders. For instance, Shen *et al.* loaded rapamycin (Rapa) into hollow mesoporous Prussian blue (HMPB) nanozymes 68. Leveraging the Fe^2+^/Fe^3+^ valence transition and multienzyme cascade activity of HMPB to scavenge ROS and simultaneously release Rapa to inhibit the mechanistic target of rapamycin (mTOR) pathway, the system synergistically blocked oxidative damage and inflammation, promoting locomotor recovery in mice with SCI.

**Figure 5:**
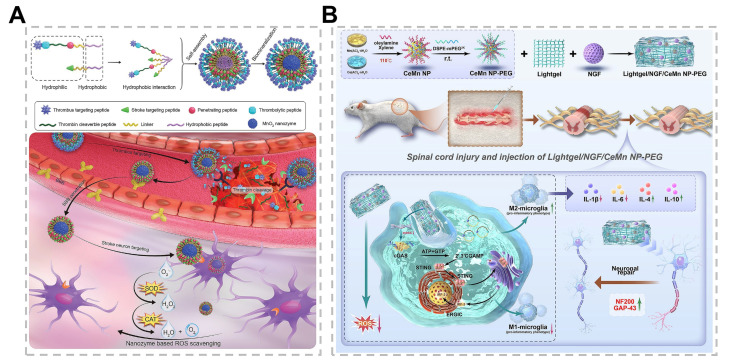
** Nanozyme-based combination therapeutic strategies: achieving neuroprotection via ROS scavenging and inflammation modulation. (A)** Thrombin-triggered PNzyme/MnO_2_ unleashes self-cascaded thrombolysis and ROS scavenging for the full-course targeted therapy of IS. Adapted with permission from 69, copyright 2023, Wiley-VCH GmbH. **(B)** A photocrosslinkable Ce–Mn/NGF hydrogel polarizes M2 microglia, eliminates ROS, and promotes neuronal regeneration via cGAS-STING inhibition for spinal cord repair. Adapted with permission from 74, copyright 2025, Gong *et al.* Abbreviations: cGAS-STING: cyclic GMP-AMP synthase-stimulator of interferon genes; IS: ischemic stroke; NGF: nerve growth factor; ROS: reactive oxygen species.

The synergistic nanozyme–drug system operates sequentially by “first clearing the microenvironment, then protecting neurons and modulating inflammation,” offering an integrated strategy to overcome the narrow thrombolysis time window, reperfusion injury, and neuronal death in IS. Building upon the “microenvironment-cooperative therapy” concept, Wang *et al.* developed a thrombin-responsive, peptide-templated MnO_2_ nanozyme (PNzyme/MnO_2_) 69. This system employs self-assembling peptides as a biomineralization scaffold for *in situ* generation of a MnO_2_ core with intrinsic SOD/CAT-mimicking cascade activity while integrating fibrin-targeting, thrombin-responsive, BBB-penetrating, and neurohoming peptide motifs. Upon thrombin activation at the thrombus site, the concealed thrombolytic peptide is exposed to achieve localized fibrinolysis, with the MnO_2_ nanozyme concurrently scavenging ROS to mitigate reperfusion injury and suppress neuroinflammation. Compared with conventional tissue plasminogen activator (tPA) combined with edaravone (Eda), which is associated with significant hemorrhagic risk and organ toxicity, this bioinspired nanoplatform demonstrated superior efficacy in reducing infarct volume, improving neurological function, and preventing neuronal apoptosis while maintaining excellent biocompatibility (Figure 5A). This strategy offers a novel approach for synergistic intervention in IS using nanozymes and functional peptides.

In addition, in order to solve the problem of poor water stability and limited functionality of traditional MOFs, Toprak *et al.* improved the water stability of CD-MOF through fluorocarbon chain surface modification 70. They introduced the hydrophobic model drug Orange OT in the process of cocrystallization, and then integrated gold nanoparticles and a lipid bilayer sequentially to build a multifunctional hybrid platform (AuNP@Lipid@CD-MOF). The system combines the hydrophobic cavity of β-cyclodextrin with the highly porous structure of MOFs, achieving a high encapsulation efficiency of 88.9%, and showing stable and sustained release characteristics under physiological conditions (pH 7.4), providing a new nanocarrier for efficient and customizable drug delivery.

These studies show that through local multi-target synergy, nanozyme–drug combination strategies can simultaneously regulate oxidative stress, inflammatory response and cell death while enhancing drug delivery. This method enhances the therapeutic effect and reduces side effects. This strategy expands the application of nanozymes in neuroprotection, and at the same time lays the theoretical foundation and provides practical guidance for precision medicine and multifunctional nanomedicine design.

### 5.2 Nanozyme–hydrogel combination

Secondary damage to the nervous system begins within minutes and can last for days to weeks; the rapid accumulation of ROS is the initiator of lipid peroxidation, mitochondrial dysfunction and DNA fragmentation, which then leads to neuronal apoptosis, axonal degeneration and myelin loss, forming a vicious cycle of "ROS damage–more ROS" 71. The existence of the BBB/BSCB makes it difficult for systemically administered drugs to reach an effective concentration at the injured area. Therefore, there is an urgent need for an intervention platform that can be injected *in situ*, provide sustained release and efficiently remove ROS.

Nanozymes combine intrinsic enzyme-like activity with low cytotoxicity, making them ideal ROS scavengers. However, their tendency to agglomerate, short half-life, difficulty crossing the BBB/BSCB, and limited ability to release drugs restrict their standalone application 19. The three-dimensional hydrophilic networks of hydrogels offer high water content, degradability, injectability, and sustained/controlled release properties. Their pore structures mimic those of extracellular matrices, enabling both the simulation of neural microenvironments and the coloading of multiple drugs. Through chemical or physical crosslinking, hydrogels can also be engineered to respond to stimuli such as ROS, pH, or temperature, achieving site-specific “smart” drug delivery 72.

On this basis, Yuan’s team designed a uniformly injectable, ROS-responsive, self-healing nanozyme hydrogel—LA/Me/Se NPs-h—in which lipoic acid (LA) and polyphenols dynamically cross-link to form a three-dimensional hydrophilic network that coloads mecobalamin (Me) and tannic acid (TA)-modified selenium nanoparticles (Se NPs) 73. Exploiting the SOD-/CAT-like activity of Se NPs, the hydrogel continuously scavenges free radicals, while its sustained-release capacity and ECM-mimetic architecture suppress particle aggregation, prolong therapeutic efficacy, and remodel the regenerative microenvironment. This achieves a multipronged, synergistic intervention that integrates antioxidant, antiapoptotic, and pro-neuroregenerative actions, offering a safe, efficient, and clinically translatable strategy for treating SCI. Furthermore, Gong *et al.* advanced this strategy by introducing growth factors onto a nanozyme–hydrogel platform (Figure 5B) 74. They coloaded cerium-manganese nanozymes (CeMn NP-PEGs) with multivalent synergistic ROS-scavenging capabilities and nerve growth factor (NGF) onto an injectable light-curable hydrogel (Lightgel), establishing a “nanozyme–hydrogel” composite system. This system leverages the three-dimensional porous structure of the hydrogel for controlled NGF release and cell colonization. Simultaneously, the SOD/CAT-like activity of CeMn NPs-PEG continuously scavenges ROS and suppresses the cGAS–STING pathway, promoting the polarization of macrophages from the proinflammatory M1 phenotype to the anti-inflammatory M2 phenotype. This synergistically improves the immune microenvironment and enhances neuronal regeneration, ultimately achieving significant recovery of motor function in animal models. A higher-order “drug–nanozyme–hydrogel–cell” four-dimensional synergistic system employs injectable GelMA as a spatiotemporal carrier, coloading selenium-doped carbon dot nanozymes (Se-CDs) and the drug FTY720; the former scavenges ROS and suppresses inflammation, while the latter blocks glial differentiation and activates endogenous neural stem cells (NSCs). Concurrently, the hydrogel anchors transplanted exogenous NSCs, providing a microenvironment that supports their survival and promotes neuron-directed differentiation. Through synergistic proliferation of endogenous and exogenous cells and synaptic reconstruction, this system achieves neural function recovery following SCI 75.

### 5.3 Nanozyme–gene combination

With the convergence of nanotechnology and bioengineering, DNA—by virtue of its programmable base-pairing specificity, rigid double-helix architecture, and excellent biocompatibility—is regarded as an ideal “building material” for constructing precise nanoscale systems 76. At the nanometer scale, DNA furnishes spatial templates whose base sites selectively coordinate metal ions, thereby directing precursor reduction and crystal growth to achieve exact control over the size, morphology, and catalytic activity of noble-metal nanozymes such as gold and platinum nanoparticles 77. Moreover, the molecular recognition capability of DNA enables specific binding to nucleic acids, proteins, small molecules, metal ions, or bacteria, transducing recognition events into catalytic signals and generating highly sensitive and selective biosensing platforms that provide robust technical support for early disease diagnosis 78. Inspired by the “enzyme–nucleic acid synergy” inherent in living systems, researchers have merged DNA’s dual capacity for “programmed recognition and interfacial-microenvironment manipulation” with nanozyme catalysis: reversible DNA adsorption/desorption enables switch-like on/off regulation of activity, while DNA sequences act as *in situ* templates for nanozyme formation, directly translating genetic information into signal output 79. This strategy provides a new generation of sensing platforms that are low-cost, rapid-response, and ultrasensitive.

In this strategy, DNA is assigned the role of an “activity valve”: (1) inhibition mode—single-stranded DNA (ssDNA) reversibly adsorbs onto the surface of nanocatalysts (*e.g.*, AuNPs, CeO_2_) via base–metal coordination or electrostatic interactions, shielding the substrate channel and thereby reducing enzyme-like activity; upon target molecule binding (complementary sequences, ATP, or metal chelators) triggering DNA desorption, catalytic sites are instantly exposed, rapidly restoring activity 80, 81. (2) Activation mode—ssDNA also functions as a dispersant or electron mediator: At an optimized DNA coating density, the DNA layer not only ceases to impede substrate entry but also enhances particle dispersion and forms molecular sieve structures. This effectively maintains catalyst site accessibility, thereby increasing the intrinsic efficiency of the nanocatalyst 80, 82, 83. Thus, the sequence information of DNA is directly translated into an “off–on” or “low–high” catalytic output, achieving a reversible, sequence-specific activity switch within minutes, offering a new route toward low-cost, rapid-response, and highly sensitive sensing platforms. For example, Mei *et al.* systematically compared the templating roles of A10, T10, C10, and G10 during the *in situ* synthesis of Pt nanozymes 77. Thymine-rich oligonucleotides (T-rich) specifically coordinate their bases with Pt^2+^/Pt^4+^, markedly accelerating precursor reduction and directing the formation of loose, porous clusters with high surface areas; consequently, POD-like activity is increased by several orders of magnitude relative to that of DNA-free controls. Conversely, sequences rich in A, C or G form a dense core–shell structure or capping layer, which prevent the substrate from entering and reduce the electron transfer efficiency, resulting in a sharp decrease in activity. This work established the sequence-dependent "bidirectional" fine-tuning of nanozyme activity for the first time.

Additionally, nanozymes can also be used as a nucleic acid delivery platform to overcome the limitations of traditional gene therapy in terms of immunogenicity, payload capacity, stability in ROS-rich environments and targeting efficiency. Nanozymes load various nucleic acid drugs (such as siRNA, mRNA, miRNA and DNA) on their surfaces through electrostatic, coordination or covalent pathways to form stable complexes 83. After entering the cell, they regulate the intracellular ROS level, promote endosomal escape and protect the nucleic acid. The subsequent release of genetic drugs can downregulate target proteins or express therapeutic proteins, thus achieving corresponding therapeutic effects 84. For example, hollow CeO_2_/Au nanozyme can efficiently load microRNA (miRNA) through electrostatic adsorption. After entering the cell through endocytosis, it clears ROS through the Ce^3+^/Ce^4+^ cycle and promotes the escape of miRNA from endosomes. Fully released miRNA can induce the downregulation of target genes by more than 60%, while achieving the synergistic effect of "ROS regulation–targeted delivery–gene therapy" 85.

### 5.4 Nanozyme–cell codelivery

Stem cell transplantation shows great prospects in tissue regeneration and repair. However, the microenvironment after damage to the central nervous system is extremely harsh: hypoxia, oxidative stress and chronic inflammation are intertwined, leading to ROS bursts, the accumulation of inflammatory cytokines (TNF-α, IL-1β, and IL-6) and insufficient blood supply. These factors put the transplanted stem cells at risk of apoptosis and functional inhibition, significantly reducing their survival rate and differentiation ability, thus seriously weakening the therapeutic effect 86. Therefore, removing excessive reactive oxygen and improving the post-injury microenvironment has become a key prerequisite for improving the efficacy of stem cell treatment. Nanozymes with multienzymatic activities have attracted much attention for their efficient and stable ROS scavenging capabilities, providing a new strategy for synergizing with stem cell transplantation and reshaping the microenvironment of the infarct zone 87.

Oxidative stress is a "key killer" that leads to a sharp decline in the efficacy of transplanted stem cells. Researchers have proposed a “nanocatalyst-empowered stem cell” strategy that uses highly active nanocatalysts to eliminate ROS and restore stem cell survival and function in oxidative stress environments. For instance, Hou *et al.* designed and synthesized negatively charged, ultrasmall Cu_5.4_O nanozymes (Cu_5.4_O-USNPs) with broad-spectrum ROS scavenging capabilities 88. These nanozymes were loaded onto adipose-derived stem cells (ADSCs) via “coincubation–spontaneous endocytosis.” The results demonstrated that compared with unmodified ADSCs, Cu_5.4_O-ADSCs exhibited significantly reduced intracellular ROS levels in oxidative stress microenvironments and markedly superior cell migration rates, vascular endothelial growth factor (VEGF) secretion levels, and angiogenesis capabilities. Building upon this foundation, Yu *et al.* further integrated nanocatalyst–nucleic acid–small molecule systems onto a single platform: CeNPs with dual SOD/CAT activity were coencapsulated with siSOX9 (which promotes neuronal differentiation) and retinoic acid (RA) within H_2_O_2_-responsive MIL-100(Fe) MOF to create CeRMS. Simple 12 h coincubation with NSCs enables efficient intracellular delivery 89. In the inflammatory microenvironment, MOF degradation releases the cargo, whereas siSOX9/RA synergistically induces NSCs to differentiate into neurons rather than into glial cells. Concurrently, CeNPs continuously scavenge ROS, providing sustained antioxidant protection for transplanted cells and their progeny.

**Figure 6:**
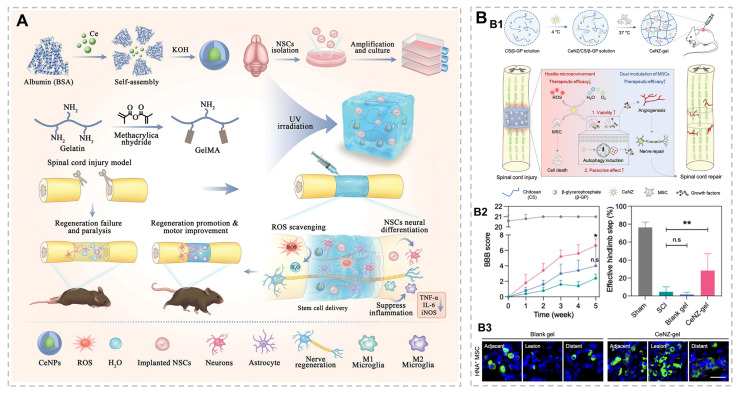
** Nanozymes combined with stem cell transplantation and smart biomaterials achieve synergistic effects on microenvironment remodeling and functional neural repair. (A)** Schematic illustration of the ability of injectable NSC-laden CeNP-Gel to promote injured spinal cord repair via ROS scavenging, M2 microglial polarization, and synergistic cooperation with NSCs. Adapted with permission from 90, copyright 2023, Wiley-VCH GmbH. **(B)** CeNZ-gel achieves efficient spinal cord repair through the dual modulation of MSCs.** (B1)** Schematic of the thermoresponsive hydrogel system: encapsulated CeNZs scavenge ROS to protect MSCs, while internalized CeNZs activate autophagy to promote angiogenic factor secretion, synergistically facilitating angiogenesis and nerve repair.** (B2)** BBB scores and hindlimb step ratios showing significant motor function recovery in the CeNZ-gel group compared with the SCI group and the blank control group.** (B3)** HNA staining validated enhanced MSC survival and distal migration in the CeNZ-gel group. Scale bar = 50 μm. Data are presented as the mean ± SD (n = 5); ^**^P < 0.01, n.s. (not significant). Adapted with permission from 92, copyright 2023, American Chemical Society. Abbreviations: BBB: Basso, Beattie, and Bresnahan locomotor rating scale; HNA: human nuclear antigen; MSCs: mesenchymal stem cells; NSC: neural stem cell; ROS: reactive oxygen species; SCI: spinal cord injury.

To address the critical bottlenecks of ROS overload and low cell survival rate after CNS injury, researchers have proposed a ternary synergistic strategy: “nanozyme ROS clearance–cell-mediated regeneration–hydrogel-delivered microenvironment.” This approach aims to establish a sustained, low-oxidative, and plastic neural-repair microplatform. For instance, Liu’s team developed an integrated “cell–nanozyme–scaffold” system: injectable GelMA hydrogels first provide mechanically matched 3D niches for NSCs, increasing survival 3.5-fold and enhancing neural differentiation efficiency 2.1-fold; embedded CeO_2_@BSA nanozymes continuously scavenge ROS, driving microglia toward the M2 anti-inflammatory phenotype, thereby suppressing oxidative stress and inflammation, and synergistically promoting axonal regeneration and remyelination, leading to rapid motor function recovery in a rat spinal cord transection model with a 5 mm defect (Figure 6A) 90. Building upon this foundation, the authors further developed an injectable hydrogel (AhCeO_2_-Gel) composed of L-arginine–loaded mesoporous hollow cerium oxide nanospheres 91. Capitalizing on the Ce^3+^/Ce^4+^ redox couple, the hydrogel scavenges excess ROS, while the released L-arginine is continuously converted to NO by inducible nitric oxide synthase (iNOS)-overexpressing microglia within the lesion. This sustained NO generation activates the Ca^2+^ influx–cAMP–PKA axis in grafted NSCs, directing their differentiation toward neurons and concurrently driving microglial polarization from the proinflammatory M1 phenotype to the reparative M2 phenotype, thereby suppressing inflammation and glial scar formation. Cotransplantation of AhCeO_2_-Gel with NSCs resulted in high cell survival, robust axonal remyelination, and rapid motor function recovery in a 5 mm full-transverse SCI rat model, suggesting the use of a “nanozyme–stem cell” combined therapeutic strategy that comprehensively reversed the inhibitory microenvironment after SCI. Additionally, Xu’s team encapsulated mesenchymal stem cells (MSCs) within a ceria-nanozyme-integrated, thermoresponsive, in situ-forming hydrogel (CeNZ-gel) 92. The hydrogel undergoes *in situ* gelation at body temperature and continuously releases ceria-based nanozymes; on the one hand, it mimics the SOD/CAT cascade to scavenge ROS, reshaping the oxidative-stress microenvironment of the SCI and markedly enhancing the survival of transplanted MSCs; on the other hand, internalized CeNZs induce autophagy, upregulating the secretion of proangiogenic and neurotrophic factors such as VEGF and Ang-1, thereby achieving dual “antioxidative–paracrine stimulation” regulation that substantially promotes nerve repair and motor function recovery (Figure 6B).

Despite the substantial therapeutic promise of nanozyme–gene and nanozyme–cell combination strategies, their clinical translation necessitates a balanced appraisal of attendant safety risks. Exogenous nucleic acids—either siRNA, miRNA, or DNA templates—carry the potential for off-target hybridization and unintended gene-network perturbation, in addition to innate immune activation via nucleic acid-sensing pathways. In stem cell codelivery systems, residual pluripotency or genomic instability of transplanted NSCs or MSCs poses a long-term risk of tumorigenicity, whereas allogeneic cellular components may trigger immunological rejection. These challenges are progressively addressable through sequence-optimized chemically modified oligonucleotides, rigorous pretransplantation differentiation and quality-control protocols, autologous cell sourcing, and immune-evasive biomaterial encapsulation. Nevertheless, future preclinical development must integrate systematic genotoxicity, immunogenicity, and long-term biodistribution studies to ensure that regenerative benefits are realized within an acceptable safety window.

### 5.5 CNS delivery strategies for nanozymes

The BBB and BSCB represent the most formidable anatomical obstacles to CNS drug delivery. Comprising tightly joined brain microvascular endothelial cells, pericytes, and astrocytic endfeet, the BBB restricts the paracellular transport of most small molecules and nearly all macromolecular therapeutics 93, 94. Consequently, systemically administered nanozymes must employ specialized strategies to access the brain parenchyma. Below, we summarize the main established paradigms for nanozyme delivery to CNS.

Intravenous administration is the most widely used route for nanozyme delivery in central nervous system diseases, but its therapeutic effect depends on the ability to cross the BBB and achieve lesion-specific accumulation. Researchers respond to this challenge through receptor-mediated transcytosis; for example, transferrin-modified MOF nanozymes use the high expression of transferrin receptors on brain microvascular endothelial cells to carry out active transendothelial transport and achieve significant enrichment in ischemic focus 95. Similarly, Zhao *et al.* used transferrin as a biomimetic mineralization template to synthesize MnO_2_ nanozyme *in situ*, and also realized BBB crossing and accumulation in ischemic foci through Tf–TfR-mediated transcytosis 96. In addition, the nanozymes coated with neutrophil-like cell membranes use the specific interaction between membrane surface integrin (LFA-1/Mac-1) and the intercellular adhesion molecule-1 (ICAM-1) on vascular endothelial cells to cross the damaged BBB and precisely home to the ischemic core 97. Furthermore, during ICH, inflammatory receptors on the neutrophil membrane (such as IL-1R and TNF-αR) interact with corresponding ligands highly expressed in the bleeding microenvironment to promote the accumulation of nanozymes in the bleeding site 98. In acute pathological conditions such as TBI, the transient opening of the BBB allows intravenously administered carbon-based nanozymes to passively extravasate into the brain parenchyma, so that this pathological window can be used to achieve cerebral distribution without additional surface modification 99.

Intranasal administration provides a non-invasive pathway for CNS delivery, which can bypass the BBB, so that the drug can directly enter the brain parenchyma through the olfactory axon and spread to the target area. Studies show that the phage-biomimetic system loaded with cerium oxide nanozyme can reach the olfactory bulb within 2 h following intranasal administration, and further spread to the ischemic core area and penumbra, exerting dual antioxidant and anti-inflammatory effects 100. In addition, Xie *et al.* confirmed that manganese oxide nanozyme can accumulate in the hippocampus and prefrontal cortex after nasal administration. By inhibiting the TLR4/NOX2 signaling pathway, these nanozymes promote the polarization of microglia to M2 phenotype, and sustained treatment for 8 weeks significantly reduced amyloid-β deposition 101. This non-invasive and repeatable strategy provides a feasible approach for long-term intervention in chronic neurodegenerative diseases such as AD.

Intracerebral direct injection (including intraparenchymal and intracerebroventricular (ICV) administration) is suitable for acute central nervous system injury or localized neurodegenerative diseases with clear foci. This method completely bypasses the blood–brain barrier and can accurately deliver nanozymes to the target area to ensure the maximum therapeutic concentration. For example, Prussian blue nanozyme (PBzyme) gradually diffuses to the substantia nigra-striatum after ICV injection. Its multienzymatic activities derived from iron multivalent conversion can effectively inhibit neuroinflammation, ultimately rescue dopaminergic neurons and restore dopamine levels 102. Although the strategy is highly invasive, it provides an irreplaceable advantage in treatment scenarios that require extremely high local drug concentrations where other routes of administration are ineffective.

In addition, intrathecal administration directly delivers nanozymes to the subarachnoid space, completely bypassing the BSCB 103. This method can achieve high local drug concentration in cerebrospinal fluid with minimal systemic exposure, and can be further combined with in-situ hydrogel implantation to establish a local sustained-release reservoir to prolong therapeutic efficacy.

Collectively, these four administration paradigms provide a set of multifunctional and complementary tools for the delivery of nanozymes in the central nervous system. Future research progress is expected to integrate multiple delivery modes into a unified nanozyme platform. By combining systemic targeting and local retention, this platform can minimize systemic exposure while maximizing the therapeutic effect.

The combined delivery platform established in this section overcomes the spatiotemporal limitations of a single catalytic intervention. However, the superiority or inferiority of any delivery strategy cannot be judged in isolation from the pathological rhythm of the disease itself. Acute CNS injury—including IS, ICH, TBI, and SCI—is characterized by ROS storms, barrier destruction and secondary inflammatory cascades from a few minutes to several hours, requiring the delivery system to have fast targeting, high-intensity clearance and short-term retention. In contrast, chronic neurodegenerative diseases such as AD and PD are characterized by low-grade oxidative stress, protein aggregation and progressive neuroinflammation that lasts from several years to decades, which require long-term stability, low toxicity, repeated dosing feasibility and lesion-specific accumulation of the delivery system. Therefore, the above delivery strategy must be carefully examined in combination with the pathological timing and anatomical barrier status of specific diseases. The following sections will systematically discuss how these engineered nanozyme platforms can be customized for the stage-specific needs of different diseases, so as to bridge the gap between proof-of-concept and precise pathological intervention.

## 6. Applications and therapeutic mechanisms of nanozymes in the treatment of CNS disorders

### 6.1 Ischemic stroke

Stroke has become the second leading cause of death in the world, with IS predominating. The key damage mechanism involves a cascade reaction triggered by reperfusion. When cerebral blood flow falls below the ischemic threshold (approximately 15 mL/(100 g·min)), neurons in the core region rapidly undergo necrosis because of energy depletion, while cells in the penumbra still possess the potential to be rescued, but they are highly susceptible to delayed apoptosis, necrosis, and inflammatory damage 104, 105. Although rt-PA thrombolysis or mechanical thrombectomy can restore blood flow, the persistent opening of the mitochondrial permeability transition pore (mPTP) and uncoupling of the electron transport chain during early reperfusion, coupled with the activation of glial NOX2, trigger a “biphasic” ROS burst, leading to severe oxidative stress 106. Excessive ROS cause mitochondrial membrane potential collapse, the release of cytochrome C and the activation of caspase-9/3-mediated apoptosis; concurrently, ROS activate caspase-1 via the NLRP3 inflammasome, inducing pyroptosis and the release of proinflammatory factors such as IL-1β and IL-18. This further amplifies NOX2/4 activity and ROS production, forming a positive feedback loop of “oxidative stress–inflammation–cell death.” Secondary injury increases the infarct volume, suppresses Wnt/β-catenin signaling to disrupt the BBB, recruits peripheral immune cells, and promotes M1 polarization of microglia. This perpetuates neuroinflammation, ultimately exacerbating neuronal damage and hindering functional recovery 107.

IS is an acute cerebrovascular event with a narrow therapeutic window. Reperfusion triggers a biphasic ROS burst via mitochondrial electron leakage and microglial NADPH oxidase (NOX2) activation in a few minutes to a few hours, initiating a self-amplifying oxidative stress–inflammation–cell death cascade. Since the BBB is basically intact in this ultra-acute phase, the nanozyme platform must achieve rapid targeted penetration (*e.g.*, inflammatory homing or receptor-mediated transcytosis) and couple high-throughput multienzyme clearance (SOD–CAT and peroxynitrite clearance) to neutralize the ROS peak. In addition to immediate antioxidant protection, these systems must also inhibit neuronal apoptosis and pyroptosis at the same time, drive microglia to polarize to M2 phenotype, and promote angiogenesis, thus bridging acute intervention and subacute repair. However, existing clinical antioxidants such as Eda are limited by single-target mechanism and poor BBB penetration, which highlights the urgent need for nanozyme systems that can integrate targeted delivery, multi-enzyme synergy and microenvironmental remodeling. The following studies show how specific material–delivery combinations respond to these stage-specific needs.

**Figure 7:**
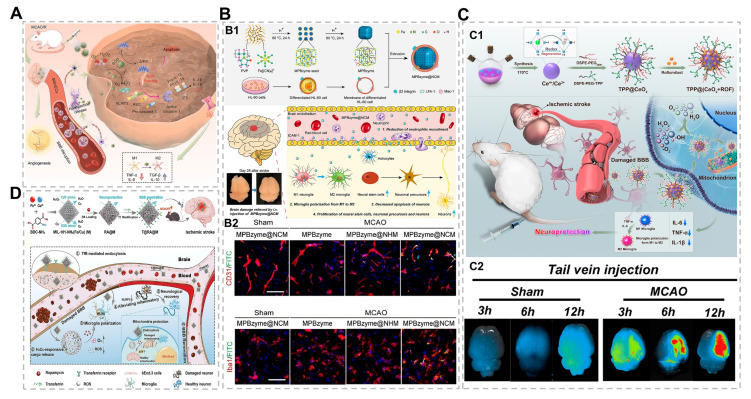
** Multifunctional nanozyme systems for IS therapy. (A)** The synergistic neuroprotective effect of PLSP@CeO_2_ nanozyme hitchhiking on neutrophils. Adapted with permission from 108, copyright 2025, Guo *et al.* **(B)** MPBzyme@NCM synthesis, targeting, and mechanism for stroke therapy. **(B1)** Synthesis and BBB penetration via integrin–ICAM-1 binding, enabling anti-inflammatory and neuroreparative therapy. **(B2)** *In vivo* fluorescence imaging of MPBzyme@NCM accumulation in the ischemic brain and its colocalization with CD31^+^ endothelial cells and Iba1^+^ microglia. Adapted with permission from 97, copyright 2021, American Chemical Society. **(C)** TPP@(CeO_2_+ROF) crosses the damaged BBB to enrich in ischemic lesions and exerts neuroprotection in MCAO rats. **(C1)** Schematic of mitochondria-targeted ROS scavenging and microglial M1→M2 polarization. **(C2)** *In vivo* imaging of lesion-directed accumulation in the ischemic hemisphere. Adapted with permission from 11, copyright 2024, American Chemical Society. **(D)** Schematic illustration of T@RA@M for targeted IS therapy: TfR-mediated BBB penetration, H_2_O_2_-triggered rapamycin release, and nanozyme ROS scavenging act synergistically for neuroprotection. Adapted with permission from 95, copyright 2024, Wiley-VCH GmbH. Abbreviations: BBB: blood–brain barrier; CD31: cluster of differentiation 31; Iba1: ionized calcium-binding adapter molecule 1; ICAM-1: intercellular adhesion molecule-1; IS: ischemic stroke; MCAO: middle cerebral artery occlusion; ROF: roflumilast; ROS: reactive oxygen species; TfR: transferrin receptor.

The neutrophil "hitchhiker" strategy uses acute inflammatory cascade to achieve blood–brain barrier penetration and focal targeting simultaneously. For example, Guo *et al.* designed a lipoic acid zwitterionic polymer nanocatalyst (PLSP@CeO_2_) 108. The sialic acid on its surface specifically binds to L-selectin on neutrophils to promote its crossing of the blood–brain barrier with the help of inflammatory chemotaxis. After reaching the lesion site, the reversible Ce^3+^/Ce^4+^ cycle gives it a SOD/CAT-like cascade activity, which induces microglia to polarize toward M2 phenotype while efficiently removing ROS, so as to achieve the regulatory cascading effect of antioxidant–anti-inflammatory–repair (Figure 7A). Continuing this paradigm, Feng *et al.* further adopted the mesoporous Prussian blue nanozyme (MPBzyme@NCM) covered with neutrophil membranes 97. By using the specific interaction between cell membrane surface integrin (LFA-1/Mac-1) and endothelial cell ICAM-1 in ischemic focus, they achieved dual enhancement through active targeting and inflammatory chemotaxis (Figure 7B).

Integrating nanozymes with functional materials or drugs to build a multifunctional system with "targeted delivery, ROS clearance and immune regulation" is a key pathway to overcome the limitations of single therapy in IS. MOFs are crucial in this strategy because they have customizable metal nodes, high drug-loading capacity and intrinsic enzyme-mimetic activity. For example, Chen *et al.* employed a SOD/CAT-mimetic bimetallic MIL-101-NH_2_(Fe/Cu) MOF as a scaffold, loaded the mTOR inhibitor rapamycin into its pores, and electrostatically coated the surface with transferrin to generate the T@RA@M nanosystem. This nanomachine exploits Tf–TfR-mediated transcytosis across the BBB, undergoes H_2_O_2_-triggered degradation within ischemic lesions, and cascades to release rapamycin, scavenge ROS, and suppress neuroinflammation (Figure 7D) 95. Moreover, Zhao’s team adopted a more streamlined biomimetic mineralization approach, directly growing MnO_2_ nanoshells *in situ* on transferrin (Tf) and coloading Eda to obtain Eda-MnO_2_@Tf (EMT) nanozymes. This system likewise crosses the BBB via the Tf–transferrin receptor 1 (TfR1) pathway, releases its payload in response to the weakly acidic, ROS-rich microenvironment, clears ROS through combined SOD/CAT/•OH-scavenging activities, and simultaneously releases Mn^2+^ for T_1_-weighted magnetic resonance imaging (T_1_-MRI), thereby enabling concurrent antioxidant therapy and imaging surveillance in IS 96.

The mitochondrial oxidative stress cascade is a core driver of ischemia–reperfusion injury, yet conventional drugs struggle to accumulate effectively within neuronal mitochondria. Liao’s team developed a mitochondrion-targeted ceria nanozyme–drug codelivery system (TPP@CeO_2_+ROF) that achieves precise mitochondrial localization through triphenylphosphine (TPP) modification 11. Using the SOD/CAT-like activity of CeO_2_ nanozyme, the system efficiently removes excessive ROS, and the incorporated PDE4 inhibitor ROF enhances the anti-inflammatory and neuroprotective effects. In the rat MCAO model, the system effectively reduced the infarct volume and inhibited neuronal apoptosis (Figure 7C).

Additionally, in order to bypass the BBB, the "nose–to–brain" pathway uses the olfactory nerve axon to enable direct delivery of inhalable nanozymes, so that intranasally administered drugs can reach the olfactory bulb, and then spread to the cortex and the ischemic penumbra, thus providing a non-invasive conduit for targeted brain delivery. Capitalizing on this, Zhu *et al.* constructed an inhalable “microbe-structured biomimetic” system in which M13 bacteriophages are covalently backpacked with Ce_0.9_Zr_0.1_O_2_ nanozymes (CZM) 100. After intranasal entry, CZM is selectively phagocytosed by M1-polarized microglia and, upon hitchhiking on inflammatory chemotaxis, accumulates in the ischemic core. Intracellular CZM harnesses its superoxide-dismutase-mimicking activity to scavenge excess ROS, suppresses oxidative stress-induced neuronal apoptosis, interrupts the ROS-driven inflammatory cascade, and repolarizes M1 microglia toward the anti-inflammatory M2 phenotype, thereby reshaping the neuroimmune microenvironment.

### 6.2 Intracerebral hemorrhage

Following ICH, mechanical compression from the hematoma causes primary injury, while blood components (*e.g.*, hemoglobin, iron ions, thrombin) and DAMPs released from damaged tissue drive secondary brain injury 109. These activities activate microglial polarization toward the proinflammatory M1 phenotype and NOX2-mediated ROS bursts. Excessive ROS directly damage neurons and oligodendrocytes via lipid peroxidation and mitochondrial dysfunction; concurrently, these processes amplify neuroinflammatory signaling, compromise BBB integrity, and promote peripheral immune cell infiltration, establishing a self-sustaining “oxidative stress–neuroinflammation” loop. Ultimately, this exacerbates the edema around the hematoma, the loss of synapses and the formation of glial scars 110. It is worth noting that hemoglobin degradation releases a large amount of free iron, which catalyzes the Fenton reaction to amplify ROS and drive ferroptosis—a pathological hallmark distinct from IS.

Unlike ischemic stroke, the BBB in the acute stage of cerebral hemorrhage has been destroyed, which makes the active barrier penetration strategy unnecessary. Instead, the nanozyme platform must quickly achieve broad-spectrum ROS removal, free iron chelation and microglial M1→M2 repolarization in the hemorrhagic focus to break the vicious cycle of oxidative stress–neuroinflammation and reduce perihematomal edema. However, traditional small molecule antioxidants have a short half-life and low cerebral accumulation, which cannot support continuous posthemorrhagic intervention. Therefore, integrating nanozyme systems with multienzyme cascade antioxidant activity, iron chelation and immune microenvironment remodeling functions is crucial for the multimodal synergistic treatment of ICH. The following studies will clarify how the nanozyme platform engineering strategy can meet these pathophysiology-specific demands.

**Figure 8:**
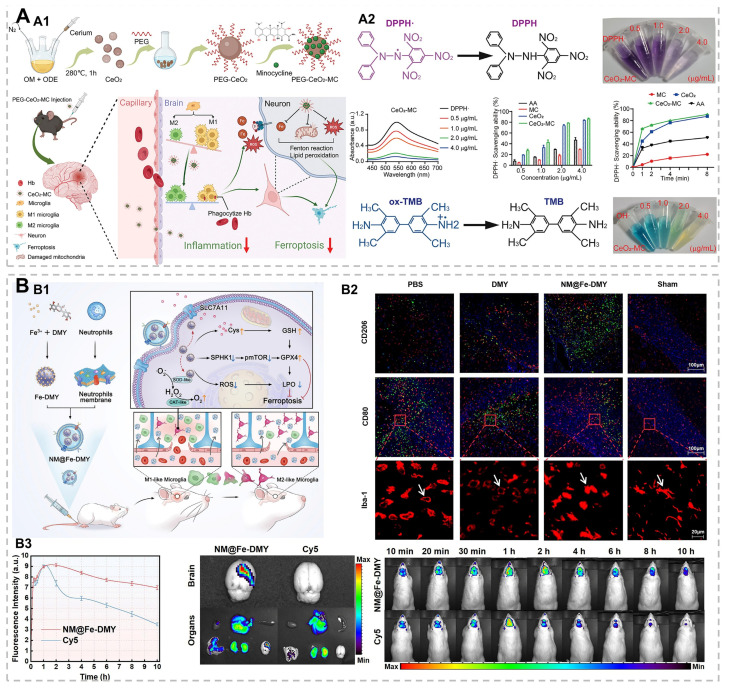
** Nanozyme-based therapeutic strategies for ICH and SAH: targeting oxidative stress, cell death, and neuroinflammation. (A1)** Multitargeted CeO_2_-MC nanoparticles protect against ICH-induced brain injury via ROS scavenging, iron chelation, microglial polarization modulation, and ferroptosis inhibition. **(A2)** CeO_2_-MC dose- and time-dependently scavenges radicals and chelates Fe^2+^, outperforming AA, MC, and CeO_2_ in terms of antioxidant capacity. Adapted with permission from 112, copyright 2024, Wiley-VCH GmbH. **(B1)** NM@Fe-DMY crosses the BBB and targets SAH lesions to alleviate early brain injury via ROS scavenging, ferroptosis inhibition, and M1-to-M2 microglial polarization. **(B2)** CD206, CD80, and Iba-1 immunofluorescence confirms the M1→M2 transition. **(B3)** Cy5-labeled NM@Fe-DMY shows time-dependent accumulation in the brain, confirming the targeting of inflammation. Adapted with permission from 114, copyright 2024, Huang *et al.* Abbreviations: AA: ascorbic acid; BBB: blood–brain barrier; CD80: cluster of differentiation 80; CD206: cluster of differentiation 206; Cy5: cyanine 5; DMY: dihydromyricetin; Iba-1: ionized calcium-binding adapter molecule 1; ICH: intracerebral hemorrhage; MC: minocycline; ROS: reactive oxygen species; SAH: subarachnoid hemorrhage.

Building on this concept, Guo *et al.* developed a laminarin-modified platinum nanozyme (Pt@LA) with a particle size of 3–4 nm 111. Its integrated SOD/CAT cascade activity increased H_2_O_2_ clearance to more than 90% within 1.5 h. Leveraging the specific binding of laminarin to Dectin-1, Pt@LA blocked the Syk/NF-κB pathway, substantially reducing M1 microglial accumulation and proinflammatory cytokine expression, improving neurological function (mNSS ↓42%) and reducing the glial scar area (↓34%) within 14 days, validating the role of the ROS-scavenging and anti-inflammatory arm. In a complementary approach targeting iron-driven pathology, Xu *et al.* proposed a synergistic drug strategy involving the loading of minocycline (MC), which possesses anti-inflammatory, antioxidant, and iron-chelating properties, onto ultrafine cerium oxide nanoparticles (CeO_2_-MC) 112. The ≈ 5 nm thermally synthesized CeO_2_ core exhibits potent radical scavenging and iron chelation, reducing free iron and ROS levels while stabilizing the mitochondrial membrane potential, thereby synergistically enhancing MC-mediated neuroprotection and inhibiting iron-induced neuronal death. In a mouse model of ICH, a single intravenous injection of CeO_2_-MC resulted in sustained accumulation at the hemorrhagic site (Figure 8A), significantly reducing hematoma volume, alleviating cerebral edema, preserving mitochondrial ultrastructure, and maintaining BBB integrity.

Membrane-biomimetic nanozymes have emerged as a versatile strategy for active targeting of hemorrhagic brain lesions across the compromised BBB. By cloaking SOD/CAT-active nanozymes with neutrophil or microglial membranes, these systems leverage inflammatory chemotaxis to achieve lesion-specific accumulation and ROS scavenging. For example, Xu* et al.* encapsulated molybdenum-based polyoxometalate (POM) nanozymes with native SOD and CAT activity in a natural chemotactic neutrophil membrane 98. The resulting POM@Mem nanodrug system can cross the damaged BBB and actively accumulate in the hemorrhagic focus, effectively reducing oxidative stress and neuroinflammation in ICH. Recently, Li *et al.* reported an ultra-small medium-entropy ruthenium single-atom nanozyme (PtRhIr/Ru SAN@M) covered with microglial membranes, which combines single-atom catalysis with medium-entropy alloys to produce excellent •OH clearance and SOD/CAT-like activity; the system penetrates the BBB, accumulates in the neuroinflammatory area, and repolarizes microglia from M1 to M2 phenotype, which significantly improves the survival rate and neurological function in the ICH model 113. Following this paradigm, Huang *et al.* constructed a neutrophil membrane-covered Fe^3+^-dihydromyricetin metal–polyphenol nanozyme, which cooperatively inhibits ROS and ferroptosis in subarachnoid hemorrhage (SAH), and drives M2-like polarization at the same time, highlighting the nanozyme–membrane bionic strategy in hemorrhagic CNS injury (Figure 8B) 114.

### 6.3 Traumatic brain injury

Traumatic brain injury (TBI) is a complex neurological disorder triggered by the instantaneous application of external mechanical force, typically resulting from traffic accidents, sports injuries, or military conflicts. Its lethal and disabling core lies not in the initial physical injury alone but in the subsequent, progressively amplified chain of secondary damage: mechanical stress triggers a surge in mitochondrial electron leakage, causing rapid increases in RONS such as O_2_^•-^, H_2_O_2_, •OH, NO, and ONOO^-^ within minutes, instantly overwhelming the buffering capacity of endogenous SOD, CAT, and GPx; lipid peroxidation products open membrane channels, allowing massive Ca^2+^ influx to activate calpain-1, which cleaves the cytoskeleton and triggers pyroptosis 115. At the same time, DAMPs activate microglial M1 polarization through the TLR/NLRP3 signaling pathway, wherein pro-inflammatory factors such as TNF-α, IL-1β and IL-6 synergize with free radicals 116. MMP-9 degrades the BBB tight junction proteins, so that peripheral immune cells can infiltrate. This forms a vicious cycle of "oxidative stress–neuroinflammation–barrier leakage", which eventually leads to irreversible neuronal loss and cognitive impairment.

This pathophysiology creates a therapeutic window distinct from that of IS: the instantaneous opening of the blood–brain barrier makes the active barrier penetration strategy unnecessary. However, the concomitant RONS burst, calcium overload and M1 microglial polarization impose composite demands on nanozyme platforms—including broad-spectrum radical scavenging, calcium homeostasis modulation and neuroinflammation suppression 117. The current treatment methods are still mainly limited to intracranial pressure management and symptomatic support, and there is a lack of targeted intervention for secondary oxidative-inflammatory cascade. Therefore, nanozyme systems that exploit this posttraumatic opening-barrier window to simultaneously neutralize RONS bursts and block secondary injury cascades are crucial for secondary-injury-modifying intervention for TBI. The following studies will clarify how specific nanozyme engineering strategies address these acute traumatic pathophysiology-specific demands. As shown by Mu* et al.*, intravenous carbogenic nanozymes use this open window to instantly capture NO, ONOO^-^, and conventional ROS while simulating the activity of multiple antioxidant enzymes. This quickly downregulates oxidative stress and neuroinflammation, thus blocking the secondary injury cascade after traumatic brain injury 99.

**Figure 9:**
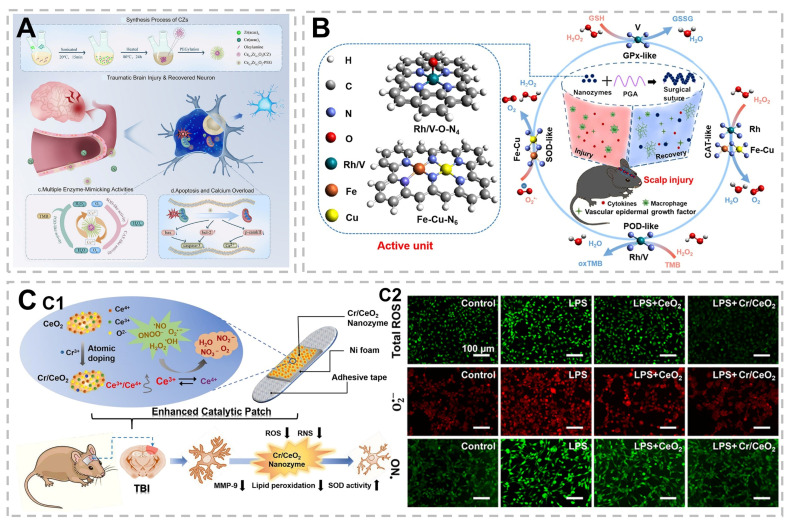
** Nanozyme-based therapeutic strategies for TBI include ROS scavenging, neural protection and tissue regeneration. (A)** Ce_0.7_Zr_0.3_O_2_ nanozymes (CZs) combined with nimodipine: multienzyme-mimetic activity, antiapoptotic effects, and calcium regulation for neuroprotection. Adapted with permission from 118, copyright 2025, Hong *et al.* **(B)** Rh/V–O–N_4_ single-atom nanozymes with superior multienzyme activities exceeding those of natural enzymes applied as regenerable bioactive sutures for brain trauma repair. Adapted with permission from 120, copyright 2022, Zhang *et al.*** (C)** Noninvasive catalytic patch based on a redox Cr/CeO_2_ nanozyme for topical TBI treatment: **(C1)** Schematic of the patch design (Cr/CeO_2_-loaded Ni foam with adhesive tape) and the RONS scavenging mechanism (↑Ce^3+^/Ce^4+^ ratio enhances SOD/CAT/GPx activity); **(C2)** *In vitro* validation showing that Cr/CeO_2_ significantly scavenges ROS (green), O_2_^•-^ (red), and NO (green) while increasing the survival of LPS-injured HT22 cells compared with those of the CeO_2_-treated and control groups. Adapted with permission from 121, copyright 2021, Zhang *et al.* Abbreviations: CAT: catalase; GPx: glutathione peroxidase; HT22: mouse hippocampal neuronal cell line; LPS: lipopolysaccharide; NO: nitric oxide; RONS: reactive oxygen and nitrogen species; ROS: reactive oxygen species; SOD: superoxide dismutase; TBI: traumatic brain injury.

In the pathophysiological process of TBI, calcium overload is a key early trigger for neuronal injury and death. Oxidative stress not only directly damages cell membranes and mitochondria, but also amplifies the calcium homeostatic imbalance, thus forming a "ROS-Ca^2+^" positive feedback loop that drives secondary cascade damage. By targeting this cross-pathway, Hong's research team integrated the powerful antioxidant nanozyme with the calcium channel blocker nimodipine to achieve the dual therapeutic effect of antioxidant protection and calcium homeostasis regulation 118. Using the ability of nimodipine to transiently modulate the permeability of the BBB, they promoted the accumulation of nanozymes in the damaged brain tissue, thus enhancing the therapeutic effect (Figure 9A).

In the process of exploring the use of nanozymes for the treatment of traumatic brain injury, researchers have optimized the performance of nanozymes through a variety of innovative strategies. For example, by creatively regulating oxidation conditions—such as oxidation duration and temperature—they optimized the size, chemical composition and electrochemical activity of coconut-derived oxidized activated carbon (cOAC) nanozymes 119. Experimental results showed that with the extension of oxidation time, the size of cOAC nanozymes gradually decreased, and their antioxidant activity was significantly enhanced. Further PEG modification significantly improved the stability and bioavailability of nanozymes in biological environments. In an *in vivo* TBI model, a single intravenous injection of PEG-modified cOAC rapidly restored cerebral blood flow perfusion. Moreover, the RhN_4_, VN_4_, and Fe–Cu–N_6_ single-atom nanozymes developed by Zhang's team achieved higher activity and stability than natural enzymes did through their unique “bilateral oxygen-linked” catalytic mechanism. These nanozymes were incorporated into bioactive sutures for use in mouse models of brain injury, significantly accelerating scalp wound healing (Figure 9B) 120. Moreover, they developed a catalytic patch based on a Cr-doped ceria (Cr/CeO_2_) nanozyme. By increasing the Ce^3+^/Ce^4+^ ratio, these nanozymes exhibit multienzyme-like activities, including POD, SOD, CAT, and GPx, and effectively scavenge various free radicals. In a mouse model of TBI, direct application of the catalytic patch to the injury site significantly accelerated wound healing, alleviated neuroinflammation, and reduced oxidative stress levels (Figure 9C) 121. Yan's research team loaded a prepared single-atom Pt/CeO_2_ catalyst onto polyacrylonitrile fibers and fixed it onto medical adhesive tape to create a nanozyme bandage. In animal experiments, a bandage was directly applied to the skin surface over the brain injury area, enabling localized treatment through the sustained release of catalytically active components 122. These findings provide new strategies and a theoretical foundation for the application of nanozymes in TBI therapy.

### 6.4 Spinal cord injury

Spinal cord injury (SCI) is severe damage to the CNS caused by traumatic and nontraumatic events and is characterized by partial or complete loss of neurological function below the injury level 123. The pathophysiological process comprises irreversible primary mechanical injury and a secondary phase—encompassing oxidative stress, inflammation, and astrocyte scar formation—that determines tissue repair and functional recovery 124. Persistent excessive ROS generation is the core trigger of secondary injury, disrupting neuronal structures and causing axonal demyelination via lipid peroxidation, DNA damage, and protein denaturation 125. Concurrently, ROS activate NF-κB and MMP-9 signaling, promoting proinflammatory factor release and glial scar formation, whereas inflammation and oxidative stress mutually reinforce M1 macrophage polarization to establish a persistent inflammatory microenvironment 126. Existing clinical interventions—including surgical decompression, corticosteroids, and anti-inflammatory agents—demonstrate limited efficacy in mitigating secondary injury, especially in promoting neural regeneration 127, 128.

In order to overcome the limitations of existing treatment methods and counter the protracted secondary cascade, the nanozyme platform needs stage-specific engineering, combining acute ROS burst inhibition with sustained microenvironmental remodeling and regenerative support. Unlike intracranial diseases, SCI is anatomically confined, and the BSCB can be bypassed by intrathecal injection or *in situ* hydrogel implantation, rendering both systemic administration and local delivery strategies feasible. Therefore, a continuous intervention framework covering acute antioxidant protection, sustained anti-inflammatory repair and the promotion of axonal regeneration must be built for the nanozyme system. The following studies will explain in detail how specific nanozyme engineering strategies can meet these biphasic pathophysiology-specific demands.

In response to the microenvironmental imbalance caused by oxidative stress, inflammation and apoptosis after SCI, Gao's team designed a cubic zirconium-doped Prussian blue nanozyme PB–Zr with a particle size of 110 nm. With the inherent multi-enzymatic activity of Prussian blue (CAT-, SOD-, and GPx-like) and the precise regulation of Zn^2+^ homeostasis by Zr^4+^, this nanozyme can simultaneously scavenge ROS, drive macrophage polarization from M1 to M2, inhibit neuronal apoptosis, and significantly restore hind-limb motor function in mice within 28 days 129.

In recent years, the nanozyme–hydrogel combination has become a research hotspot for damaged spinal cord repair. Nanozymes have enzyme-like catalytic activity, which can efficiently and stably remove RONS, thus alleviating oxidative stress. Hydrogels are characterized by injectability, self-adhesion and three-dimensional porous structure, providing physical support and a biochemical microenvironment conducive to axonal regeneration 130. After integration, these two components synergistically perform multiple functions through a single *in situ* injection—including antioxidation, anti-inflammation, nutrient delivery, and tissue bridging. This method provides a simple and efficient platform for multi-target combined treatment of SCI. For example, the zinc-pyrogallol nanozyme (PA-Zn) designed by the Chen team exhibited SOD/CAT activity. Loading PA-Zn into an injectable hydrogel yields the PA-Zn/Gel system. In a mouse T10 complete-transection model, a single local injection of 0.1 mg/kg PA-Zn/Gel significantly suppressed macrophage infiltration and NF-κB/IκBα inflammatory signaling in the lesion within two weeks, reduced the white matter cavity area, improved ventral horn neuron survival, and promoted hindlimb motor function recovery 131.

**Figure 10:**
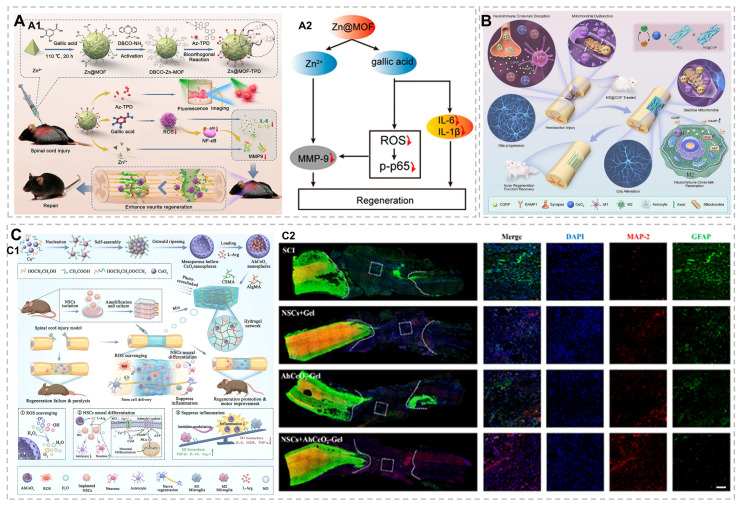
** Multifunctional nanozyme systems for injured spinal cord repair: integrating ROS scavenging, immunomodulation, and stem cell therapy. (A)** Zn@MOF-TPD nanozyme system for injured spinal cord repair: bioorthogonal synthesis of AIE-active nanozymes **(A1)** and a multimodal therapeutic mechanism integrating ROS scavenging with MMP-9 inhibition to suppress neuroinflammation and promote neural regeneration **(A2)**. Adapted with permission from 132, copyright 2024, American Chemical Society. **(B)** The NS@COP scaffold promotes axon regeneration and motor recovery after SCI via antioxidant activity, signaling, and mitochondrial protection. Adapted with permission from 133, copyright 2024, Zheng *et al.* **(C)** Schematic and *in vivo* validation of the use of AhCeO_2_-Gel for injured spinal cord repair. **(C1)** Synthesis of L-Arg-loaded AhCeO_2_ nanospheres and their incorporation into the CSMA-AlgMA hydrogel for ROS scavenging, on-demand NO release, and NSC delivery.** (C2)** MAP-2/GFAP staining of spinal cord sections at 8 weeks: the NSCs+AhCeO_2_-Gel group showed maximal neuronal formation and minimal glial scar formation. Adapted with permission from 91, copyright 2025, American Chemical Society. Abbreviations: AIE: aggregation-induced emission; GFAP: glial fibrillary acidic protein; L-Arg: L-arginine; MAP-2: microtubule-associated protein 2; MMP-9: matrix metalloproteinase-9; MOF: metal-organic framework; NO: nitric oxide; NSCs: neural stem cells; ROS: reactive oxygen species; SCI: spinal cord injury.

Metal‒organic frameworks have emerged as ideal carriers for coupling nanozyme functions because of their ultrahigh specific surface area and ease of postmodification. Zheng *et al.* incorporated aggregation-induced emission (AIE) ligands into zinc-MOF to construct the ROS-responsive nanozyme Zn@MOF-TPD. Upon triggering by peroxides at the lesion site, the framework dissociates, simultaneously releasing gallic acid and Zn^2+^. This synergistically scavenges ROS and blocks the NF-κB-MMP-9 inflammatory axis, achieving neuronal protection and myelin repair (Figure 10A) 132.

Following SCI, oxidative stress and inflammatory responses interact to create an inhibitory microenvironment that not only impedes endogenous neural repair but also significantly decreases the efficacy of exogenous therapies such as neural stem cell transplantation. To address this, Liu *et al.* incorporated L-Arg-loaded hollow mesoporous ceria nanozymes (AhCeO_2_) into an injectable, photocrosslinkable CSMA-AlgMA hydrogel to establish an AhCeO_2_-Gel system, which enables on-demand sustained NO release at iNOS-high expression sites, promoting NSC neurogenesis via the Ca^2+^-cAMP-PKA axis and inducing M2 polarization of microglia to reduce inflammation and glial scar formation (Figure 10C) 91. This hydrogel fills defects, suppresses oxidative stress, and codelivers cells and drugs, significantly enhancing neural circuit integration and motor recovery in rats with a 5 mm complete transverse SCI. Zheng *et al.* developed an intelligent nanofiber scaffold (NS@COP) by embedding enzyme-mimetic ceria nanoparticles (COPs) into polycaprolactone (PCL) nanofibers 133. The scaffold uses the three-dimensional porous structure of PCL to provide mechanical support and guide the directional migration of cells. Through in-situ anchoring, the scaffold not only prevents the agglomeration and burst release of COPs, but also improves the catalytic efficiency with its high specific surface area. In a hemisection SCI model, NS@COP significantly reduced glial scar formation, promoted myelin regeneration, and significantly enhanced the endogenous repair capacity (Figure 10B).

### 6.5 Neurodegenerative diseases: Alzheimer's disease

Alzheimer's disease (AD) is one of the most common neurodegenerative diseases among the elderly. Its main characteristics are progressive cognitive dysfunction, memory loss and behavioral abnormalities 134. Its characteristic pathological manifestations include extracellular senile plaques with Aβ deposition as the core, and intracellular neurofibrillary tangles (NFTs) formed by hyperphosphorylated tau protein aggregation 135. In terms of pathophysiological mechanisms, abnormal Aβ aggregation is believed to induce synaptic dysfunction, mitochondrial damage and oxidative stress response by activating a variety of cell surface receptors, ultimately triggering neuronal apoptosis. In addition, hyperphosphorylated tau protein disrupts the stability of microtubules, interferes with axonal transport and synaptic function, and is closely related to cognitive decline. Additionally, neuroinflammation, dysregulation of metal ion (Cu, Fe, Zn) homeostasis, glial cell dysfunction, and the transregional spread of Aβ and tau proteins in the brain all play an important role in the occurrence and progression of AD 136, 137. Notably, oxidative stress, as a common pathway of multiple pathological mechanisms, not only directly damages neurons, but also can aggravate neurodegeneration by promoting Aβ production, tau protein phosphorylation and inflammatory response, thus forming a positive feedback loop 138.

Unlike the characteristics of a sharp increase in ROS in acute CNS injury, the course of AD progresses for several years. Its core pathological characteristics include persistent low-grade oxidative stress, metal-ion dyshomeostasis and progressive deposition of misfolded proteins (Aβ and tau). This chronic pathological rhythm requires the nanozyme platform capable of repeatedly or continuously crossing the blood–brain barrier and accumulating in the hippocampus and cerebral cortex; given the integrity of the BBB structure, effective brain accumulation depends on active transcytosis. However, traditional drugs (such as cholinesterase inhibitors) can only provide symptom relief and cannot block pathological cascade or overcome barrier penetration limitations 139. Therefore, nanozyme systems integrating multi-enzyme cascade antioxidant activity, metal ion chelation and targeted Aβ/tau clearance have become the key to disease-modifying therapy 140.

For example, Luo *et al.* constructed a Ru^3+^-based nanozyme (Ru^3+^-NMOF) that catalyzes H_2_O_2_ to generate •OH, oxidizing Aβ peptide chains and increasing their hydrophilicity, thereby inhibiting Aβ aggregation and disaggregating fibrils while alleviating Aβ-induced neuroinflammation 141. Additionally, the copper (Cu)- and zinc (Zn)-based bimetallic peptide framework nanozymes (CuZn-PEP NZs) designed by Zhang *et al.* exhibit highly efficient ROS scavenging capabilities by mimicking the active site of natural copper-zinc SOD (Cu/Zn-SOD) 142. Simultaneously, the hydrogen bonds and electrostatic interactions on the CuZn-PEP NZ surface, combined with the catalytic properties of metal ions, disrupt the stable structure of the Aβ fibrils, thereby achieving fibril depolymerization.

**Figure 11:**
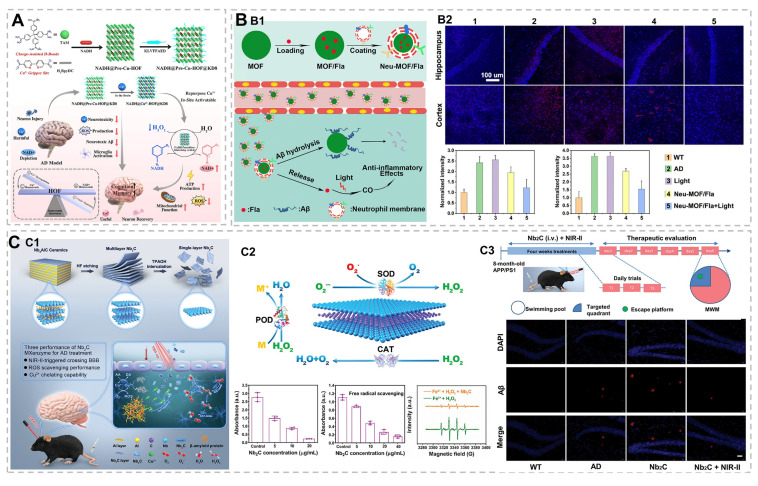
** Nanozyme-based therapeutic strategies for AD: targeting metal homeostasis, amyloid-β pathology, and oxidative stress. (A)** NADH@Pre-Cu-HOF@KD8 sequesters Cu^2+^ from Aβ-Cu^2+^ and, upon Cu^2+^ binding, exhibits *in situ*-activated NADH POD activity to restore Cu^2+^/NAD^+^ homeostasis in AD mice. Adapted with permission from 143, copyright 2025, Wiley-VCH GmbH.** (B)** Biomimetic engineering and *in vivo* validation of Neu-MOF/Fla. **(B1)** Neutrophil membrane coating enables BBB crossing and inflammatory targeting, integrating light-triggered CO release with Aβ hydrolysis to disrupt the amyloid-inflammation cycle. **(B2)** Compared with no treatment, combined photoactivation (group 5) markedly decreased the number of Aβ plaques in AD mice (group 2), reducing plaque numbers toward levels observed in wild-type controls (group 1). Scale bar, 100 μm. Adapted with permission from 145, copyright 2024, Liu *et al.* **(C)** Nb_2_C MXenzyme crosses the BBB under NIR-II photothermal stimulation to chelate Cu^2+^ and scavenge ROS via multienzyme-like activities **(C1–C2)**, thereby improving spatial memory and reducing the hippocampal Aβ burden in APP/PS1 mice **(C3)**. Adapted with permission from 144, copyright 2022, Wiley-VCH GmbH. Abbreviations: Aβ: amyloid-β; AD: Alzheimer's disease; APP/PS1: amyloid precursor protein/presenilin 1; BBB: blood–brain barrier; CO: carbon monoxide; HOF: hydrogen-bonded organic framework; MOF: metal-organic framework; NAD^+^: oxidized nicotinamide adenine dinucleotide; NADH: reduced nicotinamide adenine dinucleotide; NIR-II: second near-infrared window; POD: peroxidase; ROS: reactive oxygen species.

Metal-ion dyshomeostasis is a key driver of AD pathology. Increased cortical Cu^2+^ levels increase ROS generation, trigger the cGAS–STING axis, and promote neuroinflammation, which accelerates cognitive decline. By engineering *in situ*-activatable HOFs, Zhang *et al.* addressed this issue by using the nanozyme NADH@Pre-Cu-HOF@KD8 143. Its 2,2′-bipyridine lattice extracts Cu^2+^ from Aβ–Cu^2+^ complexes, neutralizing toxicity while endowing the particle with NADH-POD-like activity that converts NADH to NAD^+^, thereby restoring redox balance and mitochondrial function. KD8-peptide decoration mediates brain entry and lesion targeting. In 3xTg-AD mice, a single systemic course decreased the Aβ burden, attenuated oxidative stress, preserved neurons, and reversed memory deficits, suggesting a potent, metal-repurposing, and metabolically corrective anti-AD strategy (Figure 11A). Building upon this foundation, Du *et al.* utilized Nb_2_AlC ceramics as raw materials, employing HF etching to remove Al and TPAOH intercalation exfoliation to obtain single-layer Nb_2_C MXene nanosheets. These nanosheets can selectively chelate Cu^2+^, thus blocking the Aβ aggregation induced by Cu^2+^ and removing ROS through SOD/CAT/POD multienzyme activities. Their NIR-II photothermal effect can reversibly enhance the permeability of the blood–brain barrier and promote the efficient accumulation of Nb_2_C in the brain. This significantly reduces Cu^2+^-related Aβ deposition and neuronal oxidative damage, while improving cognitive function and providing good biosafety (Figure 11C) 144. This method provides a new multi-mechanism nanozyme therapeutic strategy for AD.

**Figure 12:**
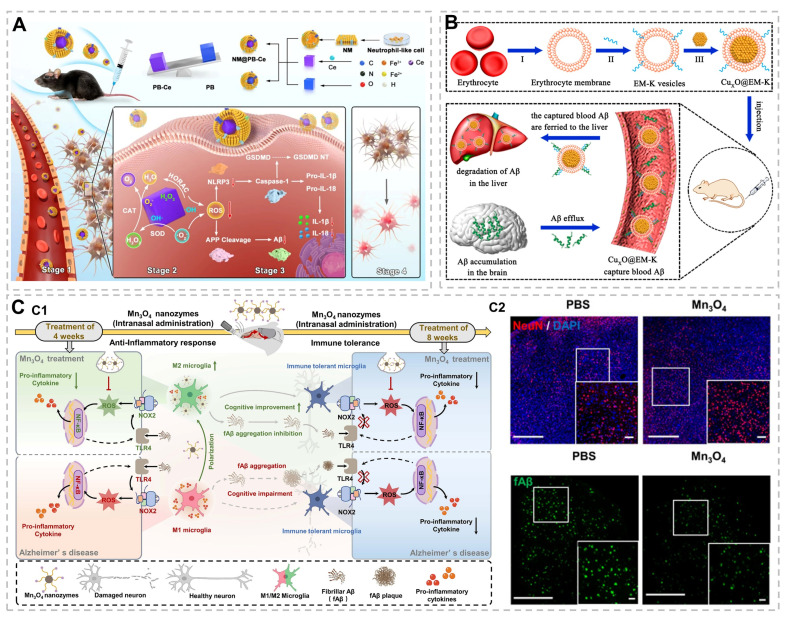
** Nanozyme-based strategies for AD: targeting neuroinflammation, pyroptosis, and amyloid-β pathology. (A)** NM@PB-Ce nanozymes cross the BBB via neutrophil membrane-mediated transport, execute the SOD-CAT-POD cascade to eliminate ROS, block the NLRP3-pyroptosis axis, and mitigate Aβ/tau pathology in AD. Adapted with permission from 146, copyright 2025, Ma *et al.* **(B)** Facile assembly of Cu_x_O@EM-K for selective peripheral Aβ sequestration and hepatic degradation, thereby inducing cerebral Aβ efflux via the “sink” effect. Adapted with permission from 147, copyright 2020, American Chemical Society. **(C)** Mn_3_O_4_ nanozymes ameliorate AD pathology via time-dependent microglial modulation. **(C1)** Intranasal delivery downregulates TLR4/NOX2, scavenges ROS, and promotes M2 polarization (4 weeks) and immune tolerance at 8 weeks, and fAβ levels decreased and cognitive performance improved. **(C2)** Prefrontal cortex at 8 weeks: NeuN^+^ neurons (left) and fAβ plaques (right). Scale bars: 100 μm (NeuN), 200 μm (fAβ); insets 50 μm. Adapted with permission from 101, copyright 2025, Xie *et al.* Abbreviations: Aβ: amyloid-β; AD: Alzheimer's disease; BBB: blood–brain barrier; CAT: catalase; fAβ: fibrillar amyloid-β; NeuN: neuronal nuclei; NLRP3: NOD-like receptor pyrin domain-containing 3; NOX2: NADPH oxidase 2; POD: peroxidase; ROS: reactive oxygen species; SOD: superoxide dismutase; TLR4: Toll-like receptor 4.

Through coordinated surface chemical modification and membrane biomimetic strategies, nanozymes can achieve blood–brain barrier crossing, inflammatory homing, microenvironment remodeling and toxic protein removal sequentially, thus systematically intervening in the complex pathological network of AD. For example, Liu *et al.* loaded the light-triggered carbon monoxide releasing molecule Fla into Zr-MOF-808 nanoparticles with uniform octahedral morphology and high specific surface area, and further cloaked them with neutrophil membranes to construct a Neu-MOF/Fla nanozyme system 145. Upon light exposure, the platform instantly releases the anti-inflammatory gas signal molecule CO at the lesion site, and at the same time uses the intrinsic hydrolase activity of MOF-808 to degrade Aβ monomers *in situ* (Figure 11B). On this basis, Ma *et al.* constructed bionic nanozyme NM@PB-Ce with cerium-doped Prussian blue (PB-Ce) as the core and neutrophil membrane (NM) as the shell 146. Leveraging the reversible oxidation of Fe^2+^/Fe^3+^ and Ce^3+^/Ce^4+^ in PB-Ce to realize SOD-CAT-POD cascade catalysis, it efficiently scavenges ROS. It relies on inflammatory homing mediated by LFA-1/Mac-1/β2 integrin on membrane surfaces, efficiently traverses the BBB and accumulates in the Aβ plaque area. Upon entering neurons, it inhibits pyroptosis via the ROS-NLRP3-ASC-caspase-1-GSDMD axis, downregulates IL-1β/IL-18, concurrently reducing Aβ aggregation and tau phosphorylation, restoring mitochondrial crista structure and dendritic spine density, and ultimately significantly improving cognitive and motor function in AD mice (Figure 12A). Ma *et al.* developed an antioxidant nanozyme, Cu_x_O@EM-K, encapsulated in erythrocyte membranes and surface modified with the Aβ-targeting peptide KLVFF 147. This biomimetic nanozyme resists protein corona formation in plasma, selectively captures Aβ, and delivers it to hepatocytes for degradation. Moreover, the multiple antioxidant enzyme-like activities of the Cu_x_O core protect the membrane from Aβ-induced oxidative damage, enabling prolonged systemic circulation. After repeated intravenous injections, 3xTg-AD mice exhibited significant reductions in Aβ levels in both the blood and the brain, along with marked amelioration of memory deficits (Figure 12B). Moreover, the HOF-based nanozyme NADH@Pre-Cu-HOF@KD8 developed by Zhang *et al.* suppresses ROS generation and neurotoxicity by sequestering Cu^2+^ from Aβ, mimics NADH POD activity to restore mitochondrial function and ATP levels, and achieves BBB crossing and lesion-targeted delivery via KD8 peptide functionalization 143. Together, these studies delineate a reproducible technical roadmap that progresses from barrier crossing through anti-inflammation and protein clearance to energy restoration, offering a modular design rationale for future preclinical optimization.

Moreover, the receptor for advanced glycation end products (RAGE) is markedly upregulated in AD, where it binds and shuttles Aβ, accelerating cerebral Aβ deposition, neuroinflammation, and neuronal injury and making RAGE an attractive therapeutic target 148. Exploiting this, Yang *et al.* constructed the nanoplatform CMOPKP to home to the AD microenvironment and reset redox balance 149. CeO_2_-PVP nanozymes with CAT-mimetic activity were first embedded into MIL-100(Fe) MOF to yield CMO; the surface was then coated with PEI and conjugated to the KLVFFAED (K8) peptide. K8 recognizes RAGE, conferring both active targeting of AD lesions and BBB penetration, thereby demonstrating a preclinical strategy for RAGE-mediated CRISPR delivery across the BBB in an AD mouse model.

Intranasal delivery uses the nose-to-brain route to transport therapeutics noninvasively and repetitively to the CNS. Taking advantage of this, Xie *et al.* engineered DSPE-PEG-Mal–decorated, water-soluble Mn_3_O_4_ nanozymes and instilled them intranasally into 5×FAD mice three times per week 101. After 4 weeks, the nanozymes reached the hippocampus and prefrontal cortex, downregulated TLR4 and NOX2, scavenged ROS, and shifted microglia from the proinflammatory M1 phenotype to the reparative M2 phenotype, markedly suppressing neuroinflammation. Continuous treatment for 8 weeks further reduced the fibrillar Aβ (fAβ) plaque load and significantly increased the neuronal NeuN signal, durably ameliorating AD-like pathology and cognitive deficits (Figure 12C).

### 6.6 Neurodegenerative diseases: Parkinson’s disease

Parkinson's disease (PD) is the second most common neurodegenerative disorder after AD, with its incidence rapidly increasing worldwide, affecting more than 10 million people globally 150, 151. The core pathological feature of PD is the progressive degeneration and death of dopaminergic neurons in the nigrostriatal pathway, leading to a significant reduction in striatal dopamine (DA). These pathological alterations result in characteristic clinical manifestations such as bradykinesia, resting tremors, rigidity, speech changes, loss of spontaneous movement, sleep disturbances, constipation, and depression 152.

The pathogenesis of PD involves the interplay of multiple pathological pathways—including oxidative stress, α-syn aggregation, and neuroinflammation—that collectively drive disease progression. Excessive ROS accumulation within mitochondria disrupts intracellular redox homeostasis, damaging dopaminergic neurons and impairing dopamine synthesis 153, 154. Abnormal conformation and aggregation of α-syn represent another core pathological feature of PD. Under pathological conditions, α-syn misfolds and aggregates into neurotoxic Lewy bodies, which not only directly injure neurons but also propagate transneuronally in a prion-like manner along neural circuits 155. Concurrently, sustained activation of the NLRP3 inflammasome in microglia releases inflammatory mediators that intensify the local inflammatory microenvironment and perpetuate a vicious cycle of neurodegeneration 154.

Similar to AD, PD is also a chronic neurodegenerative disease; however, its core pathology is not amyloid plaque deposition, but the progressive degeneration of dopaminergic neurons in substantia nigra. Driven by persistent mitochondrial oxidative stress, pathological α-syn aggregation and prion-like transneuronal propagation, and persistent microglial NLRP3 inflammasome activation, this chronic pathological process requires the nanozyme platform to chronically traverse the BBB, and enriched in the nigrostriatal region, thus providing continuous mitochondrial protection, ROS scavenging and α-syn aggregation inhibition. At present, although alternative therapies based on levodopa can alleviate motor symptoms, they cannot prevent neurodegenerative progression or restore dopaminergic neuronal function 156. Therefore, the nanozyme system that integrates cascade antioxidant activity, NLRP3 inflammasome inhibition, α-syn aggregation blocking and dopaminergic neuroprotection has become the key to disease-modifying therapy. The following studies will explain how specific nanozyme engineering strategies can cope with these chronic degenerative demands.

**Figure 13:**
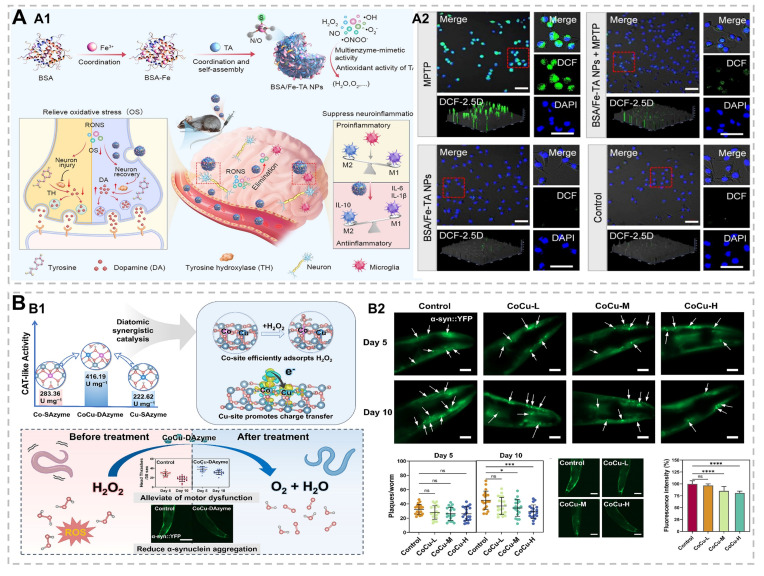
** Single-atom and dual-atom nanozymes for PD: BBB penetration, microglial modulation, and α-syn pathology modulation. (A)** BSA/Fe-TA single-atom nanozymes: **(A1)** BSA/Fe-TA nanozymes, which are fabricated via Fe^3+^-tannic acid coordination on BSA, penetrate the BBB to concurrently scavenge RONS and promote M2-like microglial polarization in neurons and microglia for PD therapy. **(A2)** BSA/Fe-TA nanozymes eliminate MPTP-induced intracellular ROS in SH-SY5Y neurons, as demonstrated by DCF fluorescence imaging. Adapted with permission from 157, copyright 2025, American Chemical Society. **(B)** CoCu dual-atom nanozymes: **(B1)** CoCu-DAzyme harnesses Co/Cu synergy to increase CAT activity and mitigate Parkinsonian phenotypes in *C. elegans*. **(B2)** CoCu-DAzyme dose-dependently inhibited α-syn aggregation and reduced total α-syn expression in *C. elegans* PD models. Adapted with permission from 158, copyright 2024, American Chemical Society. Abbreviations: α-syn: α-synuclein; BBB: blood–brain barrier; BSA: bovine serum albumin; *C. elegans*: *Caenorhabditis elegans*; CAT: catalase; DCF: 2',7'-dichlorofluorescein; MPTP: 1-methyl-4-phenyl-1,2,3,6-tetrahydropyridine; PD: Parkinson's disease; RONS: reactive oxygen and nitrogen species; ROS: reactive oxygen species.

**Figure 14:**
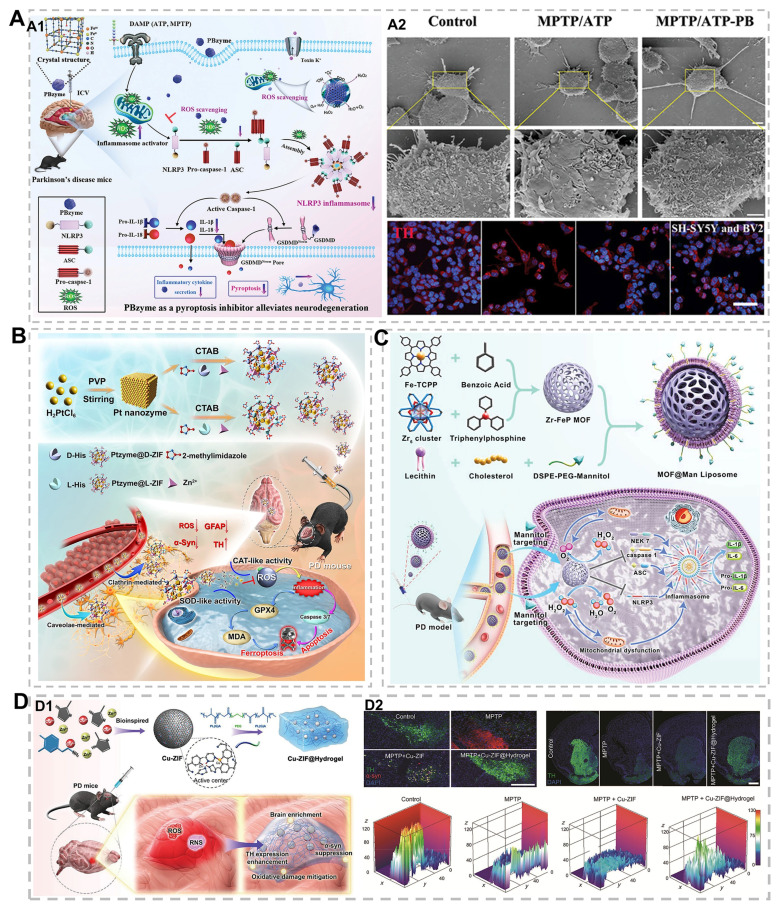
** Nanozyme-mediated modulation of neuroinflammation and cell death pathways for PD therapy. (A)** Schematic and cellular validation of PBzyme as a pyroptosis inhibitor in PD:** (A1)** intracerebroventricular delivery enables ROS scavenging and suppression of microglial NLRP3 inflammasome-mediated pyroptosis;** (A2)** PBzyme suppresses MPTP/ATP-triggered microglial pyroptosis (SEM) and rescues dopaminergic neurons in coculture (TH immunofluorescence), which is consistent with NLRP3 inflammasome inhibition. Adapted with permission from 102, copyright 2022, Wiley-VCH GmbH. **(B)** Chiral nanozyme-mediated antiparkinsonian therapy: Ptzyme@D-ZIF traverses the BBB via clathrin/caveolae-mediated transcytosis, exerting anti-Parkinsonian effects through ROS scavenging and suppression of apoptosis and ferroptosis. Adapted with permission from 159, copyright 2023, Jiang *et al.* **(C)** MOF@Man liposome nanozymes block the NLRP3 inflammasome and curb oxidative stress and neuroinflammation for PD therapy. Adapted with permission from 160, copyright 2023, Wiley-VCH GmbH. **(D)** Bioinspired Cu-ZIF nanozyme integrated into a thermosensitive PLGA-PEG-PLGA hydrogel for PD treatment.** (D1)** Injectable Cu-ZIF@Hydrogel bypasses the BBB, accumulates in the brain, and scavenges RONS to protect dopaminergic neurons.** (D2)** Immunofluorescence and 3D analysis revealed that Cu-ZIF@Hydrogel suppressed microglial activation and proinflammatory cytokine expression in the substantia nigra. Adapted with permission from 163, copyright 2023, Tsinghua University Press. Abbreviations: 3D: three-dimensional; ATP: adenosine triphosphate; BBB: blood–brain barrier; MPTP: 1-methyl-4-phenyl-1,2,3,6-tetrahydropyridine; NLRP3: NOD-like receptor pyrin domain-containing 3; PD: Parkinson's disease; RONS: reactive oxygen and nitrogen species; ROS: reactive oxygen species; SEM: scanning electron microscopy; TH: tyrosine hydroxylase.

In order to break the vicious cycle of oxidative stress and neuroinflammation in PD, researchers are developing nanozyme systems with strong antioxidant and neuroprotective functions through multi-enzyme cascade catalysis and atomic structure optimization. For example, He *et al.* used BSA as a template to self-assemble FeCl_3_ and tannic acid into a BSA/Fe-TA nanozyme with a particle size of about 15 nm 157. In this material, the iron center achieves atomic dispersion and shows a variety of enzyme activities such as SOD, CAT, POD, and GPx, thus realizing the broad-spectrum scavenging of RONS. At the same time, the surface properties of BSA enable it to efficiently cross the BBB through the pathways mediated by caveolae and albumin receptors, thereby increasing enrichment in neurons and microglia. This activity promotes synergistic antioxidant and anti-inflammatory regulation, promotes the polarization of microglia to M2 anti-inflammatory phenotype, and improves the motor and cognitive function of PD model mice (Figure 13A). In addition, Wang *et al.* used the atomic layer coordination strategy to construct a cobalt-copper dual-atom nanozyme (CoCu-DAzyme) 158. By anchoring Co and Cu atoms on the γ-AlO(OH) carrier, they achieve high-efficiency CAT-like catalytic activity. Density functional theory calculations show that the Co site mainly promotes the adsorption of H_2_O_2_, while the Cu site promotes charge transfer. Their synergistic interaction significantly reduces the reaction energy barrier, thereby accelerating the conversion of H_2_O_2_ into H_2_O and O_2_ (Figure 13B). Additionally, Ma’s team optimized the hydrothermal synthesis of PVP-coated Prussian blue nanozymes (PBzymes) with uniform particle sizes, endowing them with SOD-, CAT-, and POD-like activities for efficient ROS scavenging. Upon intracerebroventricular injection, PBzyme diffused into the nigrostriatal region, significantly inhibiting the NLRP3–caspase-1–GSDMD pathway, rescuing TH^+^ neurons and restoring dopamine levels (Figure 14A) 102.

Morphology and crystal-facet modulation have also been demonstrated to be powerful tools for increasing the catalytic efficiency of nanozymes. Singh *et al.* systematically compared the catalytic profiles of Mn_3_O_4_ nanozymes with different morphologies and reported that owing to their high specific surface area (97.7 m^2^/g), unusually large pore size (8.9 nm) and reversible Mn^2+^/Mn^3+^ redox couples, these nanozymes markedly outperform cubes (M1), polyhedra (M2), hexagonal plates (M3) and flakes (M4) in terms of SOD, CAT and GPx activity. In an MPP-induced PD cellular model, Mn_3_O_4_ nanoflowers efficiently scavenged ROS, restored neurite loss, inhibited caspase-3/7 activation, and provided robust cytoprotection to SH-SY5Y cells, offering a new structural optimization paradigm for single-component nanozymes 37. Moreover, Jiang *et al.* transplanted the concept of “chiral recognition” into a nanozyme-delivery platform and engineered D-histidine–functionalized zeolitic imidazolate frameworks that entrap ultrasmall platinum nanozymes (Ptzyme@D-ZIF) (Figure 14B) 159. Leveraging concerted clathrin- and caveolae-mediated endocytosis–transcytosis, the resulting D-chiral formulation extends the plasma half-life to 6.72 h (versus 0.64 h for its L-enantiomer) and elevates brain accumulation to 2.20% ID/g (versus 1.42% ID/g). In MPTP-induced PD mice, Ptzyme@D-ZIF robustly scavenges ROS and MDA; suppresses the NF-κB cascade and the downstream expression of TNF-α, IL-6, and IL-1β; halts neuronal apoptosis and ferroptosis; reinstates TH^+^ neuron density in the substantia nigra; and restores striatal dopamine levels, thereby restoring motor coordination and spatial memory. Li *et al.* constructed a hierarchical MOF nanozyme system (Figure 14C) 160. Hollow Zr-FeP MOFs, self-assembled from Fe-TCPP and Zr_6_ clusters, exhibit SOD/CAT cascade antioxidant activity and are encapsulated within a mannose-liposome (MOF@Man Liposome) shell that mediates receptor recognition and BBB traversal. After membrane fusion with BBB cells, the MOF core is released into the brain parenchyma. In MPTP-induced PD mice, the platform markedly reduced ROS levels and α-syn aggregation, restored substantia nigra TH^+^ neuron counts and striatal dopamine content, suppressed NLRP3 inflammasome activation, and improved motor behavior.

Additionally, PD-associated chronic neuroinflammation continuously releases chemokines that recruit peripheral macrophages to cross the BBB and home to the lesioned substantia nigra. Leveraging this inflammation-guided homing capacity, researchers have engineered macrophages to engulf therapeutic nanozymes to construct a “cell–nanozyme” hybrid delivery system. This strategy exploits the inflammatory chemotaxis and barrier-crossing capacity of macrophages to achieve precise enrichment of nanozymes within the brain. For instance, Batrakova *et al.* constructed a “macrophage–nanozyme” delivery system in which CAT was self-assembled with the cationic–hydrophilic block copolymer PEI-PEG into 60–100 nm nanozymes, which were rapidly taken up by BMM (≈ 30 µg/10^6^ cells within 40–60 min) and released active enzymes for ≥ 4–5 days 161. In MPTP-intoxicated mice, intravenously reinfused nanozyme-loaded BMMs doubled the brain ^125^I-labeled CAT content compared with free nanozymes (≈ 0.6% vs 0.3% injected dose), markedly scavenging the hydrogen peroxide generated by microglia activated with nitrated α-syn or TNF-α, thereby validating its antioxidant, targeted therapeutic potential. Zhao *et al.* further demonstrated that nanozymes loaded into BMMs specifically accumulate in affected brain regions and are continuously redistributed from peripheral organs to inflamed lesion sites over time 162.

In recent years, a nanozyme–hydrogel coupling strategy has been highly anticipated: the localized phase transition of the hydrogel prolongs nanozyme retention and decreases systemic toxicity, while the nanozyme maintains high catalytic activity within the hydrogel microenvironment, resulting in “one injection, sustained ROS scavenging”. Accordingly, Fan *et al.* embedded biomimetically mineralized Cu-ZIF nanozymes into a PLGA-PEG-PLGA thermosensitive hydrogel, thereby constructing an injectable Cu-ZIF@Hydrogel system 163. The carrier is a free-flowing sol at room temperature and undergoes a phase transition to form a stable gel at approximately 37 °C, thereby extending the residence time of Cu-ZIF at the injection site. The hydrogel microenvironment preserves its SOD/CAT-like activity for the continuous elimination of RONS. *In vitro*, the mitochondrial function of MPP^+^-injured SH-SY5Y cells was restored; *in vivo*, the construct bypassed the BBB to accumulate at the lesion, reduced neuroinflammation, restored TH^+^ neurons and striatal dopamine levels, and significantly ameliorated motor impairments in PD mice (Figure 14D).

Overall, current nanozyme research has evolved from “single-enzyme mimicry” toward “multienzyme coupling–smart delivery–pathological pathway targeting,” achieving systemic protection ranging from ROS scavenging to inflammation intervention and even apoptosis regulation. These advancements support further translational evaluation of nanozyme-based PD interventions (Table 3).

The disease-specific analyses presented above demonstrate that nanozyme platforms have achieved precise adaptation to the divergent pathological timelines of acute injuries versus chronic degenerative diseases. However, disease-specific efficacy validation simultaneously generates distinct translational constraints: acute therapeutic regimens impose stringent requirements for batch-to-batch consistency and acute toxicity profiling; chronic long-term administration exposes systematic gaps in long-term retention, degradation kinetics, and immunogenicity; and disparate barrier penetration and lesion accumulation strategies correspond to divergent regulatory pathways and clinical scalability challenges. Therefore, the establishment of disease-stage suitability is not the translational endpoint but the starting point for identifying translational bottlenecks. The following sections integrate these clinically emergent constraints—originating from specific material–delivery–disease combinations—into a unified translational feasibility assessment framework.

### 6.7 Synthesis: pathophysiology-driven design principles across CNS disorders

The rational development of CNS nanozymes does not follow a universal template; rather, it is governed by a hierarchical logic in which disease pathophysiology dictates the selection and integration of each design module.

At the foundational level, all six diseases exhibit catalytic convergence. SOD/CAT-mimicking cascade activity constitutes the essential antioxidant core, reflecting the shared primacy of RONS dysregulation in both acute injury and chronic neurodegenerative diseases. This enzymatic baseline is generalizable across indications. Similarly, the secondary anti-inflammatory regulation mediated by the transformation of the M1 to M2 phenotype of microglia emerges as a convergent downstream endpoint driven by the shared ROS–NLRP3/NF-κB–cytokine axis. Therefore, broad-spectrum ROS scavenging remains the indispensable catalytic foundation for any CNS nanozyme platform.

From the structural level, barrier penetration differences become a key determining factor. The integrity state of the BBB/BSCB imposes disease-specific restrictions on the delivery strategy. ICH and TBI use acute BBB opening caused by mechanical tear or blood component erosion, so that systemically administered nanozymes can be directly passively extravasated to the lesion. In contrast, the relatively intact IS and chronic neurodegenerative diseases (AD, PD) in the early reperfusion phase require active transcytosis—whether receptor-mediated, cell-mediated or intranasal pathway—to achieve effective brain accumulation; Some platforms can also realize passive accumulation through the lesion-specific microenvironment. Restricted by the existence of the BSCB, SCI uniquely benefits from the *in situ* hydrogel implantation loaded with nanozymes, which bypasses barrier restrictions while providing mechanical support. Therefore, the penetration strategy is dictated not by the nanozyme material alone but by the specific barrier pathology of the target disease.

At the modular level, the combination strategy is gradually upgraded according to the complexity and temporal demands of the disease. For acute RONS burst suppression in early TBI and ICH, simple nanozyme monotherapy is sufficient. The addition of anti-inflammatory or chelating drugs addresses secondary inflammatory amplification, as shown in the cerium oxide loaded with minocycline in hemorrhage. For pathological conditions accompanied by significant tissue defects and regeneration needs, the combination strategy needs to integrate neuroregenerative modules to reach the highest integration tier: in SCI, the nanozyme–hydrogel system combined with neural stem cell transplantation and nerve growth factor delivery can bridge structural defects and promote axon regeneration; in PD, the nanozyme platform must synergistically protect dopaminergic neurons and inhibit the aggregation of α-syn to preserve nigrostriatal dopaminergic function. Against this background, the core role of nanozymes has been extended from simple RONS scavenging to the coupling of microenvironmental remodeling and regeneration and repair. This hierarchical model shows that the nanozyme platform should not be regarded as a fixed formulation, but should be regarded as an adaptive system that can integrate disease-specific functional modules according to pathological stages and regeneration needs.

Overall, these cross-pathology patterns support a pathophysiology-first design philosophy: rather than selecting materials on the basis of intrinsic catalytic properties, rational CNS nanozyme development should proceed from the systematic analysis of disease temporal profiles, barrier integrity, dominant reactive oxygen/nitrogen (RONS) species and regeneration needs.

## 7. Clinical translation challenges and future perspectives

Although nanozymes have shown strong neuroprotective effects in preclinical models, their clinical translation still faces interrelated obstacles such as biosafety, pharmacokinetics, blood–brain barrier penetration and manufacturing scalability. The catalytic persistence underlies therapeutic efficacy but may pose long-term safety risks: long-term systemic exposure may cause off-target oxidative damage to healthy endothelium and mononuclear phagocyte system (MPS) organs, while protein-corona formation can change biological distribution and trigger immunogenicity. What makes the problem more complicated is that many nanozymes targeting the central nervous system exceed the renal filtration threshold (≈ 5.5 nm), resulting in unresolved uncertainties regarding hepatic sequestration, MPS accumulation, and long-term organ deposition that current short-term rodent toxicology studies fail to address 166. Therefore, rigorous preclinical research must incorporate comprehensive ADME (absorption, distribution, metabolism, excretion) characterization, standardized activity assays, and chronic toxicity tracking beyond the existing single-dose paradigm. In addition to spatial off-target biodistribution, the catalytic characteristics of nanozymes themselves have also caused biosafety concerns at the mechanism level: because low-level RONS serve as indispensable signaling molecules in synaptic plasticity, neurovascular coupling and stem cell fate regulation, excessive or poorly targeted scavenging risks oversuppressing physiological redox tone and blunting adaptive pathways (*e.g.*, Nrf2 and HIF-1α); at the same time, non-specific catalytic activity in non-lesion tissues may disturb the systemic redox homeostasis and compromise repair 167.

The species differences in the structure of the BBB and the efflux transporter activity constitute a major translational hurdle in the process of preclinical-to-clinical extrapolation; therefore, for advanced brain targeting strategies, reliable validation in large-animal or non-human-primate models is still lacking 168. At the same time, the therapeutic effect of nanozymes is highly sensitive to particle size, valence state and surface chemical properties, but the batch-to-batch consistency in the process of large-scale production is not well controlled. Therefore, industrialization requires processes that comply with good manufacturing practice (GMP) and are equipped with integrated quality control indicators to uniformly cover physicochemical characterization, catalytic standardization and biological safety readouts—and these goals remain largely unrealized for most reported systems.

Looking to the future, advancing clinical translation requires coordinated progress in two interrelated aspects. First, standardized preclinical frameworks must replace the current fragmented situation: unified animal models (including non-human primates for BBB validation), biomarker-aligned efficacy endpoints (ROS profiles, inflammatory cytokines, neurorepair proteins and multimodal imaging), and adaptive early-phase trial designs with longitudinal biomarker tracking 23, 169. Secondly, regulatory-industrial alignment must address nanomaterial-specific chemistry, manufacturing, and controls (CMC) hurdles—batch-to-batch reproducibility, sterilization validation, and *in vivo* degradation/clearance evidence—through proactive engagement with the U.S. Food and Drug Administration (FDA) and China's National Medical Products Administration (NMPA) to define nanozyme-specific investigational new drug (IND) pathways. Substantial breakthroughs in these aspects are crucial for nanozymes to transition from proof-of-concept studies to first-in-human trials for CNS disorders.

In addition, the integration of nanozyme technology and clinical neuroengineering represents an emerging translational frontier. Implanted bioelectronic systems—including brain–machine interfaces (BMIs), closed-loop neuromodulation devices and deep brain stimulation (DBS) electrodes—have great potential in restoring nerve function; however, their long-term implantation inevitably triggers foreign-body reactions, manifested as microglial activation, astrocytic hypertrophy and oxidative stress 170. These processes together drive the formation of fibrotic encapsulation and glial scar formation (typically 50–100 μm thick by week 6), progressively increasing the electrode impedance and reducing signal fidelity 171. Recent proof-of-concept studies show that integrating nanozymes with catalytic activity into electrode coatings provides a bioactive interface strategy: compared with traditional metal electrodes, nanozyme-modified electrodes can achieve significantly lower electrochemical impedance, higher charge storage capacity, and strong radical-scavenging activity, thus attenuating peri-implant oxidative stress and reducing neuronal damage around implants 172. Although long-term coating stability under electrical stimulation and standardized device–tissue integration remain to be addressed, this convergence already positions nanozymes as core functional modules in next-generation bioelectronic therapeutics, well beyond their traditional role as standalone drugs.

## 8. Conclusion

Overall, nanozymes—artificially programmable biomimetic enzyme platforms—offer unique, multitarget and network-level advantages for treating CNS injuries and neurodegenerative diseases. Here, we systematically review the progress in the use of various nanozymes in rodent models of IS, ICH, TBI, SCI, PD and AD, focusing on their combined therapeutic effects, including the following: scavenging excess ROS, relieving mitochondrial dysfunction, suppressing neuroinflammation, reshaping the immune microenvironment, and promoting angiogenesis and neuroregeneration. Compared with traditional small-molecule antioxidants or natural enzyme preparations, nanozymes can enhance CAT, SOD, and POD-like activities by tuning their composition and structure; moreover, the use of doping, defect engineering, and surface modification further enables the precise control of catalytic activity, targeting capacity, and pharmacokinetics, thereby creating a local microenvironment that favors neuroprotection and regeneration. Nevertheless, most evidence still derives from short-term rodent studies; systematic validation of long-term functional outcomes, neural circuit reconstruction, cognitive improvement, and cross-species reproducibility is lacking. The optimal intervention time, dosing frequency, and combination strategies for the acute, subacute, and chronic stages remain to be defined, and clinically relevant safety windows, dose–response relationships, and posttreatment effects have not yet been mapped. Thus, while their potential is promising, a rational and cautious stance is warranted.

Future research on nanozymes in the field of central nervous system diseases is expected to advance along the roadmap, starting from precision design, through mechanism dissection and standardized evaluation, and moving towards clinical exploration. At the material-and-structure level, disease-specific pathological microenvironments (such as high ROS levels, elevated glutathione levels, low pH, metal ion imbalance, etc.) can be exploited to construct smart nanozymes with adaptive responsiveness and on-demand release functions; integration with neurotrophic factors, small molecule drugs, nucleic acid therapeutics or extracellular vesicles will enable multimodal, synergistic interventions. At the mechanism level, single-cell omics, multi-omics integration and high spatiotemporal-resolution imaging technologies should be used to systematically map how nanozymes remodel cell metabolism, polarize immune cells, regulate glial scar formation and coordinate axon regeneration, providing a solid theoretical foundation for rational application. For translation, indications with acceptable safety profile and mature delivery routes (*e.g.*, mild-to-moderate IS or focal lesions in surgically accessible sites) should be selected for small-scale, phased exploratory trials, and multimodal imaging and fluid biomarkers should be combined to achieve multi-dimensional efficacy and safety readouts. Cross-fertilization with digital medicine, radiomics, and neuromodulation should also be pursued, exploring combinations such as “nanozyme + cell therapy” or “nanozyme + brain–computer-interface/neuromodulation”. Rather than a single universal therapy, nanozymes are poised to become a modular, highly versatile platform within future multidisciplinary treatment ecosystems. Once safety, controllability, and scalability are achieved, the use of nanozymes could shift CNS therapy from traditional symptomatic management strategies to disease-modifying and function-restoring strategies.

## Figures and Tables

**Table 1:** Classification of nanozymes by composition structure.

Metal-based nanozymes	Metal oxide-based nanozymes	Carbon-based nanozymes	Porous framework-based nanozymes
Gold nanoparticles (AuNPs)	Iron oxide nanoparticles (Fe_3_O_4_)	Carbon nanotubes (CNTs)	ZnO quantum dot-anchored zeolitic imidazolate framework-8 (ZIF-8) nanozymes
Silver nanoparticles (AgNPs)	Co-Doped Fe_3_O_4_ Nanozyme	Carbon quantum dots (CQDs)	Se-MOF-derived dual-Fe-atom nanozymes (Fe_2_NC@Se-MOF)
Platinum nanoparticles (PtNPs)	Cerium oxide nanoparticles (CeO_2_)	Graphene	Zinc-MOF nanozymes
Palladium nanoparticles (PdNPs)	Copper oxide nanoparticles (CuO/Cu_2_O)	Carbogenic nanozymes	Hollow Zr-FeP MOF nanozymes
Gold nanoclusters (AuNCs)	Manganese oxide nanoparticles (MnO_2_)	Selenium-doped carbon dots (Se-CDs)	MIL-101-NH_2_(Fe/Cu) MOF nanozymes
Mesoporous-silica-encapsulated gold nanoparticles (EMSN-AuNPs)	Cerium-zirconium oxide nanozymes (Ce_0.9_Zr_0.1_O_2_, Ce_0.7_Zr_0.3_O_2_)	Graphitic carbon nitride (g-C_3_N_4_)	Zirconium-based MOF-808 nanoparticles (Zr-MOF-808)
Palladium-core platinum-shell nanoplates (Pd@Pt nanoplates)	Cerium-manganese nanozymes (CeMn NPs)		NADH-loaded pre-Cu-chelating hydrogen-bonded organic framework with KD8 peptide (NADH@Pre-Cu-HOF@KD8)

Abbreviations: All abbreviations are defined *in situ* within the table cells.

**Table 2:** Nanozyme-based combination therapeutic strategies.

Combined therapeutic strategy	Combined modality	Experimental model	Core function	Diseases	Ref.
Nanozyme–Drug Combination	Rapamycin-loaded hollow mesoporous Prussian blue nanozyme (RHPAzyme)	C57BL/6J mouse T11-T12 contusion model	High rapamycin loading was achieved in hollow mesoporous Prussian blue nanozymes equipped with MMP-responsive ACPP for SCI lesion targeting, creating an integrated "antioxidant–anti-inflammatory" nanosystem.	SCI	68
Nanozyme–Drug Combination	AuNPs@CD-MOF + Orange OT (model drug) + DPPC (lipid stabilizer)	*In vitro* cellular assays	Sequential surface assembly enables concurrent high drug loading, NIR photothermal elevation, POD-like catalysis and sustained release.	—	70
Nanozyme–Drug Combination	PNzyme/MnO_2_ + GRPAK thrombolytic peptide + multitargeting peptides	Mouse suture MCAO and rat autologous-thrombus embolic stroke models	15 nm peptide-templated MnO_2_ nanozyme combining thrombin-triggered thrombolytic peptide exposure with intrinsic SOD/CAT cascade antioxidant activity	IS	69
Nanozyme–Hydrogel Combination	Zero-valent selenium nanozymes + lipoic acid/mecobalamin dual-dynamic hydrogel	Adult female ICR mice with T10 spinal cord contusion	A single injectable selenium-nanozyme hydrogel broadly scavenges ROS, upregulates SOD-positive reactive astrocytes, suppresses caspase-3, preserves myelin and fully restores SCI-mouse locomotion (BMS 9) within 15 days.	SCI	73
Nanozyme–Hydrogel Combination	Lightgel/NGF/CeMn NP-PEG composite + CeMn NP-PEG nanozyme + NGF	Adult female SD rat T8–9 spinal cord contusion model	A single *in situ* injection of the composite hydrogel sustainably scavenges ROS, promotes M2 polarization, reduces inflammation, and enhances axonal regeneration.	SCI	74
Nanozyme–Hydrogel Combination + NSCs	Selenium-doped carbon dots + FTY720 + Gelatin methacryloyl + NSCs	Adult female SD rats with T9 transection	Single-shot synchronized “ROS scavenging → anti-inflammation → NSC-to-neuron differentiation → scar suppression” closed-loop repair.	SCI	75
Nanozyme–Hydrogel Combination	ZnO-decorated ZIF-8 nanozymes+ injectable PVA-Alg hydrogel	C57BL/6J mice with contusion at the T9/T10 spinal segment	ZnO-ZIF8 nanozymes enable NIR/acid-triggered Zn^2+^ release, loaded into injectable PVA-Alg hydrogel for photothermal-antioxidant scaffold.	SCI	66
Nanozyme–Gene Combination	DNA-encoded Pt nanozymes	*In vitro* assays	DNA-sequence-encoded mono-repeat oligos (A10/T10/C10/G10) template *in situ* Pt-nanozyme synthesis for sequence-tunable activity.	—	77
Nanozyme–Cell Codelivery	CeNPs–siSOX9–RA@MIL-100(Fe) coincubated with NSCs	11-month-old 3×Tg-AD mice	Using recyclable SOD/CAT-active CeNPs as a built-in antioxidant shield, the study protects newborn neurons from secondary oxidative damage after NSC transplantation.	AD	89
Nanozyme–Cell Codelivery	Cerium Oxide Nanoparticles + Gelatin Methacryloyl + NSCs	5 mm transection rat SCI model	A “nanozyme-NSC-hydrogel” system was built to scavenge ROS and regulate microglial polarization, enhancing NSC survival and neural regeneration for rapid SCI recovery.	SCI	90
Nanozyme–Cell Codelivery	L-Arg-loaded hollow mesoporous CeO_2_ nanozymes + CSMA-AlgMA hydrogel + GFP-labeled NSCs	SD rats with T10 complete 5 mm transection	AhCeO_2_-Gel exploits the high-ROS/iNOS SCI milieu to scavenge ROS and trigger on-demand L-Arg→NO, delivering lesion-targeted NO without systemic toxicity and steering engrafted NSCs toward neurons.	SCI	91
Nanozyme–Cell Codelivery	Ceria nanozyme-integrated thermoresponsive *in situ* forming hydrogel + MSCs	Complete spinal cord transection model in rats	CeNZ-gel enhances MSC survival and promotes vascular and neural repair through ROS scavenging and CeNZ-induced autophagy, which boosts VEGF/Ang-1/TGF-β1 secretion.	SCI	92

Abbreviations: 3×Tg-AD: triple-transgenic Alzheimer's disease; A10: adenine 10-mer oligonucleotide; ACPP: activatable cell-penetrating peptide; AD: Alzheimer's disease; Alg: alginate; Ang-1: angiopoietin-1; AuNPs: gold nanoparticles; C10: cytosine 10-mer oligonucleotide; CAT: catalase; CD: cyclodextrin; DNA: deoxyribonucleic acid; DPPC: 1,2-dipalmitoyl-sn-glycero-3-phosphocholine; FTY720: fingolimod; G10: guanine 10-mer oligonucleotide; GelMA: gelatin methacryloyl; GFP: green fluorescent protein; GRPAK: a thrombolytic peptide released upon thrombin cleavage; ICR: Institute of Cancer Research; iNOS: inducible nitric oxide synthase; IS: ischemic stroke; L-Arg: L-arginine; MCAO: middle cerebral artery occlusion; Me: mecobalamin; MIL-100(Fe): iron-based MIL-100 metal-organic framework; MMP: matrix metalloproteinase; MOF: metal-organic framework; MSC: mesenchymal stem cell; NGF: nerve growth factor; NIR: near-infrared; NO: nitric oxide; NPs: nanoparticles; NSC: neural stem cell; PEG: polyethylene glycol; PNzyme: peptide-templated MnO_2_ nanozyme; POD: peroxidase; PVA-Alg: polyvinyl alcohol-alginate; RA: retinoic acid; ROS: reactive oxygen species; SCI: spinal cord injury; SD: Sprague-Dawley; SOD: superoxide dismutase; T10: thymine 10-mer oligonucleotide; TGF-β1: transforming growth factor-β1; VEGF: vascular endothelial growth factor; ZIF-8: zeolitic imidazolate framework-8.

**Table 3:** Nanozyme applications and mechanisms in CNS disorders treatment.

Disease	Nanozyme	Enzyme-like Activity	Animal Model	Administration Route	Therapeutic Effect	Ref.
IS	Poly (lipoic acid)-Sialic acid-Protocatechualdehyde@CeO_2_ (PLSP@CeO_2_)	SOD, CAT	MCAO model in rats	Intravenous injection	Infarct volume↓from 41.7% to 9.6%; TNF-α/IL-1β/IL-6↓; preserved BBB integrity.	108
IS	Neutrophil-like cell membrane-coated mesoporous Prussian blue nanozyme (MPBzyme@NCM)	SOD, CAT	Transient middle cerebral artery occlusion (tMCAO) in mice	Intravenous injection	Neuronal apoptosis↓; neurogenesis↑; neurological function↑; 28-day survival 50→91%; shifts microglia M1→M2.	97
IS	Tf@RA@MIL-101-NH_2_(Fe/Cu) MOF nanozyme	SOD, CAT	MCAO/R in C57BL/6 mice	Intravenous injection	ROS↓; neuronal apoptosis↓; neuroinflammation↓; infarct volume↓from 41.5% to ≈ 14.4%; preserved BBB integrity.	95
IS	Eda-MnO_2_@Tf (EMT)	SOD, CAT	MCAO model in rats	Intravenous injection	ROS↓; TNF-α/IL-1β/IL-6↓; infarct volume↓ (41.7%→9.6%)	96
IS	TPP-Ceria Nanozymes (CeO_2_) +ROF	SOD, CAT	MCAO model in rats	Intravenous injection	Infarct volume↓; neuronal apoptosis↓; neuroinflammation↓; mitochondrial function↑; neurological function↑; protected BBB.	11
IS	Ce_0.9_Zr_0.1_O_2_ nanozymes (CZM)	SOD	MCAO model in rats	Intranasal administration	Infarct volume↓; oxidative stress↓; neuronal apoptosis↓; shifts microglia M1→M2; motor function↑.	100
ICH	Laminarin-modified ultrasmall platinum nanozyme (Pt@LA)	SOD, CAT	Collagenase VII-induced ICH in rats	Single stereotactic intracerebral injection	TNF-α/IL-6/IL-1β↓; MDA (malondialdehyde)↓; GFAP scar↓; preserved neurons; blocked M1 microglia; hematoma↓; neurological function↑.	111
ICH	Minocycline-loaded cerium oxide nanoparticles (CeO_2_-MC)	SOD, CAT	Autologous blood-induced ICH in C57BL/6 mice	Intravenous injection	Brain edema↓; preserved BBB integrity; neuroinflammation↓; ferroptosis↓; ameliorated long-term neurobehavioral deficits.	112
ICH	Neutrophil membrane-coated POM nanoclusters (POM@Mem)	SOD, CAT	Collagenase IV-induced ICH in mice	Intravenous injection	IL-1β/IL-6/TNF-α mRNA↓; intracellular ROS↓; neuronal proliferation↑.	98
SAH	Neutrophil membrane-coated Fe-DMY nanozyme (NM@Fe-DMY)	SOD, CAT	Endovascular puncture SAH model in rats	Intravenous injection	72 h survival↑; Garcia score (48–72 h) ↑; shifts microglia M1→M2. ferroptosis↓; neuroinflammation↓.	114
TBI	Carbogenic Nanozyme	SOD, CAT	Mice TBI models	Intravenous injection	RONS↓; MMP-9↓; oxidative stress↓; SOD↑; neuronal apoptosis↓; BBB permeability↓; neurological function↑.	99
TBI	Ultrasmall Ce_0.7_Zr_0.3_O_2_ nanozymes (CZs)	SOD, CAT, POD	CCI-induced TBI in adult C57BL/6 mice	Intravenous injection	BBB integrity↑; cerebral edema↓ (79.6% → 73.2%); oxidative stress↓; neuroinflammation↓; neuronal apoptosis↓; cognitive and motor functions↑.	118
TBI	Single-atom nanozymes (RhN_4_, VN_4_, Fe-Cu-N_6_)	POD, CAT, SOD, GPx	Fluid-percussion TBI in C57BL/6 mice	Surgical suture	Neuroinflammation↓ (IL-6 ↓70%; IL-1β↓65%; TNF-α↓60%); angiogenesis↑; oxidative stress↓; neurological function↑; scalp healing↑.	120
TBI	Cr-doped CeO_2_ (Cr/CeO_2_) nanozyme	SOD, CAT, GPx	Fluid-percussion TBI in C57BL/6 mice	Noninvasive catalytic patch	Neuroinflammation↓ (GFAP↓, Iba-1↓, MMP-9↓); lipid peroxidation↓(MDA↓); SOD↑; spatial learning/memory↑; wound closure↑.	121
SCI	Prussian blue–Zr hybrid nanozyme (PB–Zr)	CAT, SOD, GPx	T9-T10 contusion SCI in C57BL/6J mice	Intraperitoneal injection	ROS↓; MDA↓, GSH/SOD↑; IL-6↓; shifts microglia M1→M2; neuronal apoptosis↓; PC12 survival and migration↑; neuron integrity↑; motor function↑; cavities↓.	129
SCI	Zinc pyrogallol nanozyme (PA-Zn)	SOD, CAT	T10 complete transection of SCI in C57BL/6 mice	*In situ* hydrogel implantation at T10	Motor function↑; neuroinflammation↓; neuron survival↑; lesion area↓; synapse↑; reparative scar↑.	131
SCI	Tellurium nanozyme	SOD, CAT	T9 contusion in female ICR mice	*In situ* hydrogel implantation	Oxidative stress↓; neuroinflammation↓; neuronal apoptosis↓; neural regeneration↑; motor function↑.	164
SCI	LA/Me/Se nanoparticle hydrogel (LA/Me/Se NPs-h)	SOD, CAT	T10 contusion SCI in female ICR mice	*In situ* hydrogel implantation	Motor function↑; lesion cavity↓; neuronal apoptosis↓; axonal regeneration↑; demyelination↓.	73
SCI	Zn-MOF decorated with aggregation-induced emission dye TPD (Zn@MOF-TPD)	SOD, CAT	T10 contusive SCI in female ICR mice	Intraperitoneal injection	Oxidative stress↓; neuroinflammation↓; MMP-9 expression↓; NSC proliferation and differentiation↑; glial scar↓; protected neurons and myelin sheaths; motor function↑.	132
SCI	ZnO-ZIF-8 functionalized hydrogel (ZnO-ZIF8@H)	SOD	T9/T10 contusive SCI in C57BL/6J mice	*In situ* hydrogel implantation	Oxidative stress↓; neuroinflammation↓; neuronal apoptosis↓; ferroptosis↓; neural regeneration↑; motor function↑.	66
SCI	L-Arg-loaded hollow mesoporous cerium oxide nanozyme (AhCeO_2_)-Gel	SOD, CAT	T10 complete transection (5 mm) in SD rats	*In situ* hydrogel implantation	ROS↓; neuronal differentiation↑; axon regrowth↑; motor function↑; glial scar↓	91
SCI	Cerium Oxide Nanoparticles (COPs)	SOD, CAT	Hemisection SCI in rats	Implantation of NS@COP scaffold	Axon regeneration↑; glial scar↓; mitochondria function↑; motor function↑; shifts microglia M1→M2.	133
AD	Ruthenium-functionalized nanoscale MOF (Ru^3+^-NMOF)	POD	*C. elegans* CL2120 (expressing Aβ-GFP)	Oral feeding (on NGM plates)	Oxidative stress↓; inhibited Aβ aggregation; disaggregated existing plaques; paralysis↓.	141
AD	NADH-loaded pre-Cu-chelating HOF-KD8 (NADH@Pre-Cu-HOF@KD8)	POD	3×Tg-AD transgenic mice	Intraperitoneal injection	ATP↑; ROS↓; Aβ plaque↓; restored cerebral NAD^+^/NADH ratio; microglial activation↓; hippocampal synaptic transmission↑; memory deficits↓.	143
AD	Nb_2_C MXene-based nanozyme (MXenzyme)	SOD, CAT, POD	APP/PS1 double-transgenic mice	Intravenous injection + NIR-II irradiation	Aβ plaque↓; neuroinflammation↓; oxidative stress↓; cognitive function↑	144
AD	Neutrophil-membrane-coated MOF-808 (Neu-MOF/Fla)	Peptide-bond hydrolysis	3×Tg-AD transgenic mice	Intravenous injection	TNF-α↓; IL-1β↓; Aβ plaque↓; neuroinflammation↓; microglia CD16^+^→CD206^+^; crosses BBB and homes to inflamed brain regions↑; cognition↑.	145
AD	TPP-modified Cu_2-x_Se nanoparticles (Cu_2-x_Se-TPP NPs)	SOD, CAT	Aβ_42_-icv-injected C57BL/6J mice	Intravenous injection	Aβ plaque↓43%; oxidative damage↓; neuronal survival↑; shifted microglia from M1 to M2; proinflammatory cytokines↓; anti-inflammatory↑; spatial memory↑; nest behavior↑.	165
AD	Neutrophil membrane-coated cerium-doped Prussian blue (NM@PB-Ce)	SOD, CAT, POD	Aβ1–42 oligomer-induced AD mouse model	Intravenous injection	Neuroinflammation↓; Aβ plaque↓; tau phosphorylation↓; pyroptosis↓; enhanced BBB penetration and mitochondrial protection; cognitive and motor function↑.	146
AD	KLVFF-erythrocyte membrane-coated Cu_x_O nanozyme (Cu_x_O@EM-K)	CAT, SOD, GPx	3×Tg-AD transgenic mice	Intravenous injection	Peripheral and brain Aβ burden↓; memory deficits rescued.	147
AD	Ceria–polyvinylpyrrolidone nanoparticles (CeO_2_-PVP)	SOD, CAT	3×Tg-AD transgenic mice	Intravenous injection	ROS↓; Nrf2-mediated redox balance restoration; hippocampal neuronal loss↓; cognitive performance↑.	149
AD	Mn_3_O_4_ nanozyme	SOD	5×FAD transgenic mice	Intranasal instillation	M2 microglial polarization↑, neuroinflammation ↓; Aβ plaque load ↓, neuronal rescue.	101
PD	Bovine serum albumin–Tannic acid–iron nanoparticles (BSA/Fe-TA NPs)	SOD, CAT, POD, GPx, APx, TPx	MPTP-induced PD mice	Intravenous injection	Oxidative stress↓; neuroinflammation↓; tyrosine hydroxylase (TH) levels↑; motor function↑.	157
PD	Co-Cu dual-atom nanozyme (CoCu-DAzyme)	SOD, CAT	Transgenic *C. elegans* NL5901	Oral feeding (on NGM plates)	ROS ↓; α-syn expression↓19%; oxidative stress↓.	158
PD	Mn_3_O_4_ nanoflowers (Mnf)	SOD, CAT, GPx	Cells	Cell study	Oxidative damage↓; neuronal apoptosis↓; neurite loss↓.	37
PD	Pt nanozymes embedded in D-chiral ZIF (Ptzyme@D-ZIF)	SOD, CAT	MPTP-induced male C57BL/6 mice	Intravenous injection	ROS↓; α-syn↓; TH neurons↑; neuroinflammation↓; neuronal apoptosis↓; ferroptosis↓; motor and memory function↑	159
PD	Zr-FeP MOF in mannitol-liposome (MOF@Man Liposome)	SOD, CAT	MPTP-induced male C57BL/6 mice	Intravenous injection	ROS↓30%; α-syn↓67%; BBB penetration↑; TH^+^ neurons↑; DA/DOPAC↑; SOD↑; GSH/GSSG↑.	160
PD	BMM-loaded nanozyme	CAT	MPTP/6-OHDA/LPS-induced C57BL/6 mice	Intravenous injection	Neuroinflammation↓; nigrostriatal degeneration↓; BBB crossing↑; targeted delivery to affected brain regions↑.	162
PD	Cu-ZIF-8-loaded thermosensitive hydrogel (Cu-ZIF@Hydrogel)	SOD	MPTP-induced PD mice	*In situ* gelling hydrogel injection	Neuroinflammation↓; TH^+^ neuron↑; striatal dopamine↑; alleviated behavioral and pathological symptoms.	163

Abbreviations: 3×Tg-AD: triple-transgenic Alzheimer's disease; 5×FAD: five familial Alzheimer's disease; 6-OHDA: 6-hydroxydopamine; Aβ: amyloid-β; AD: Alzheimer's disease; AhCeO_2_: L-arginine-loaded hollow mesoporous cerium oxide nanozyme; APP/PS1: amyloid precursor protein/presenilin 1; APx: ascorbate peroxidase; ATP: adenosine triphosphate; BBB: blood–brain barrier; BMM: bone marrow-derived macrophage; *C. elegans*: *Caenorhabditis elegans*; CAT: catalase; CCI: controlled cortical impact; CD: cluster of differentiation; CoCu-DAzyme: cobalt-copper dual-atom nanozyme; Cu_2-x_Se-TPP NPs: copper selenide-triphenylphosphine nanoparticles; Cu-ZIF@Hydrogel: Cu-ZIF-8 nanozyme-loaded hydrogel; CZM: Ce_0.9_Zr_0.1_O_2_ nanozymes; DA: dopamine; DOPAC: 3,4-dihydroxyphenylacetic acid; EMT: edaravone-MnO_2_@transferrin; GFAP: glial fibrillary acidic protein; GFP: green fluorescent protein; GPx: glutathione peroxidase; GSH: glutathione; GSSG: oxidized glutathione; HOF: hydrogen-bonded organic framework; Iba-1: ionized calcium-binding adapter molecule 1; ICH: intracerebral hemorrhage; ICR: Institute of Cancer Research; ICV: intracerebroventricular; IL: interleukin; IS: ischemic stroke; M1: pro-inflammatory phenotype; M2: anti-inflammatory phenotype; MCAO/R: middle cerebral artery occlusion/reperfusion; MDA: malondialdehyde; MMP-9: matrix metalloproteinase-9; MOF: metal-organic framework; MOF@Man Liposome: mannose-liposome-encapsulated metal-organic framework; MPTP: 1-methyl-4-phenyl-1,2,3,6-tetrahydropyridine; MXene: two-dimensional transition metal carbide, nitride, or carbonitride; NAD^+^: oxidized nicotinamide adenine dinucleotide; NADH: reduced nicotinamide adenine dinucleotide; NGM: nematode growth medium; NIR-II: second near-infrared window; NM@Fe-DMY: neutrophil membrane-coated Fe^3+^-dihydromyricetin; NPs: nanoparticles; Nrf2: nuclear factor erythroid 2-related factor 2; NS@COP: nanofiber scaffold with ceria nanoparticles; NSC: neural stem cell; PC12: rat pheochromocytoma cell line; PD: Parkinson's disease; POD: peroxidase; POM: polyoxometalate; PVP: polyvinylpyrrolidone; ROS: reactive oxygen species; RONS: reactive oxygen and nitrogen species; Ru^3+^-NMOF: ruthenium-functionalized nanoscale metal-organic framework; SAH: subarachnoid hemorrhage; SCI: spinal cord injury; SD: Sprague-Dawley; SOD: superoxide dismutase; TBI: traumatic brain injury; TH: tyrosine hydroxylase; tMCAO: transient middle cerebral artery occlusion; TNF-α: tumor necrosis factor-α; TPx: thiol peroxidase; TPP: triphenylphosphine; ZIF: zeolitic imidazolate framework; α-syn: α-synuclein.

## Abbreviations

Aβ: amyloid-β
AD: Alzheimer's disease
ADSCs: adipose-derived stem cells
AIE: aggregation-induced emission
ATP: adenosine triphosphate
NCs: nanoclusters
NPs: nanoparticles
BBB: blood–brain barrier
BMIs: brain–machine interfaces
BSA: bovine serum albumin
BSCB: blood–spinal cord barrier
CAT: catalase
CDs: carbon dots
CMC: chemistry, manufacturing, and controls
CNS: central nervous system
CNTs: carbon nanotubes
COFs: covalent organic frameworks
CQDs: carbon quantum dots
DA: dopamine
DALYs: disability-adjusted life years
DAMPs: damage-associated molecular patterns
DBS: deep brain stimulation
DFT: density functional theory
DNA: deoxyribonucleic acid
DNN: deep neural network
DSF: disulfiram
EC: Enzyme Commission
Eda: edaravone
fAβ: fibrillar amyloid-β
FDA: Food and Drug Administration
GBR: gradient boosting regression
GDY: graphdiyne
GOx: glucose oxidase
GPx: glutathione peroxidase
GSHOx: glutathione oxidase
H_2_O_2_: hydrogen peroxide
HMPB: hollow mesoporous Prussian blue
HOF: hydrogen-bonded organic framework
HRP: horseradish peroxidase
ICH: intracerebral hemorrhage
ICAM-1: intercellular adhesion molecule-1
ICV: intracerebroventricular
IgG: immunoglobulin G
IND: investigational new drug
iNOS: inducible nitric oxide synthase
IS: ischemic stroke
LA: lipoic acid
LLM: large language model
MCAO: middle cerebral artery occlusion
MC: minocycline
Me: mecobalamin
miRNA: microRNA
MOF: metal-organic framework
MPS: mononuclear phagocyte system
MSC: mesenchymal stem cell
mPTP: mitochondrial permeability transition pore
NGF: nerve growth factor
NFT: neurofibrillary tangle
NMPA: National Medical Products Administration
NOX2: NADPH oxidase 2
NSC: neural stem cell
O_2_^•-^: superoxide anion
•OH: hydroxyl radical
ONOO^-^: peroxynitrite
OXD: oxidase
PBzyme: Prussian blue nanozyme
PCMs: phase change materials
PCL: polycaprolactone
PD: Parkinson's disease
PEI: polyethyleneimine
POD: peroxidase
POM: polyoxometalate
RA: retinoic acid
RAGE: receptor for advanced glycation end products
Rapa: rapamycin
RONS: reactive oxygen and nitrogen species
ROS: reactive oxygen species
SAH: subarachnoid hemorrhage
SAzyme: single-atom nanozyme
SCI: spinal cord injury
SDT: sonodynamic therapy
SHAP: Shapley additive explanations
SOD: superoxide dismutase
ssDNA: single-stranded DNA
TA: tannic acid
TBI: traumatic brain injury
TCPP: tetracarboxyphenylporphyrin
Tf: transferrin
TfR: transferrin receptor
T1-MRI: T1-weighted magnetic resonance imaging
TMB: 3,3',5,5'-tetramethylbenzidine
tPA: tissue plasminogen activator
TPP: triphenylphosphine
US: ultrasound
VEGF: vascular endothelial growth factor
α-syn: α-synuclein
